# GZMK⁺CD8⁺ T Cells Induce Acinar Cell Senescence and Salivary Secretion Impairment in Sjögren's Syndrome via Mitochondrial Dysfunction and TMEM16A Chloride Channel Disruption

**DOI:** 10.7150/ijbs.134478

**Published:** 2026-10-02

**Authors:** Maosheng Chai, Yufei Xie, Xin Cong, Chong Ding, Pan Wei, Peiru Zhou, Binbin Li, Hong Hua

**Affiliations:** 1 Department of Oral Medicine, Peking University School and Hospital of Stomatology & National Center for Stomatology & National Clinical Research Center for Oral Diseases & National Engineering Research Center of Oral Biomaterials and Digital Medical Devices, Beijing, China.; 2 Department of Physiology and Pathophysiology, Peking University School of Basic Medical Sciences, State Key Laboratory of Vascular Homeostasis and Remodeling, Beijing, China.; 3 Central Laboratory, Peking University School and Hospital of Stomatology, National Center for Stomatology, National Clinical Research Center for Oral Diseases, National Engineering Research Center of Oral Biomaterials and Digital Medical Devices, Beijing Key Laboratory of Digital Stomatology, NHC Key Laboratory of Digital Stomatology, NMPA Key Laboratory for Dental Materials, Beijing, China.; 4 Department of Oral Pathology, Peking University School and Hospital of Stomatology & National Engineering Research Center of Oral Biomaterials and Digital Medical Devices, Beijing, China.; 5 Research Unit of Precision Pathologic Diagnosis in Tumors of the Oral and Maxillofacial Regions, Chinese Academy of Medical Sciences (2019RU034), Beijing, China.

**Keywords:** Sjögren's Syndrome, GZMK⁺CD8⁺ T cells, acinar cell senescence, salivary secretion impairment, mitochondrial dysfunction, TMEM16A

## Abstract

Immune cell-mediated damage to acinar cells plays a central role in Sjögren's syndrome (SS) pathogenesis, with T-cell recruitment being critical in the early stages. In this study, single-cell RNA sequencing was employed to generate transcriptomic profiles of labial gland tissues from SS patients, focusing on GZMK⁺CD8⁺ T cells and their correlation with SS clinical pathology. The role of GZMK⁺CD8⁺ T cells in inducing acinar cell senescence and secretory dysfunction was investigated by co-culturing SMG-C6 acinar cells with GZMK⁺CD8⁺ T cells and recombinant granzyme K. Mechanistically, GZMK induces oxidative stress and mitochondrial dysfunction, thereby promoting senescent phenotypes in acinar cells. Additionally, GZMK-mediated downregulation of TMEM16A contributes to reduced chloride transport and salivary secretion dysfunction. This study provides novel insights into the immune-epithelial crosstalk in SS progression by revealing the relationship between GZMK⁺CD8⁺ T cells and acinar cells. This pathogenic immune-epithelial interaction may offer a promising therapeutic strategy to mitigate SS progression in the early stages and restore salivary secretion in SS patients.

## Introduction

Sjögren's syndrome (SS) is a representative autoimmune disease that primarily affects exocrine glands, such as the salivary and lacrimal glands, leading to progressive glandular damage and dysfunction 1. With an estimated prevalence of 0.3% to 0.7% in the Chinese population, it affects approximately 4.5 to 10 million individuals, positioning it as the second most prevalent autoimmune disease 2-4. The typical clinical manifestations of SS include xerostomia, xerophthalmia, fatigue, and arthralgia 5. Approximately one-third of patients develop systemic involvement, such as interstitial lung disease, hepatic dysfunction, and renal complications 6, 7. Alarmingly, SS patients are 15 to 20 times more likely to develop lymphoma than the general population, posing a substantial threat to their health and survival 8-10.

SS predominantly affects middle-aged and elderly women, typically presenting around the age of 55, with a male-to-female ratio ranging from 1:9 to 1:14 3, 5. The prevalence in the elderly population reaches as high as 3% to 4%, which is 5 to 8 times higher than in younger age groups 11, 12. Driven by the global trend of population aging, the prevalence of SS is still on the rise. More concerningly, older SS patients not only experience more severe xerostomia and xerophthalmia, but also face a significantly higher risk of serious complications 13-16. These clinical observations highlight that senescence plays a critical role in driving SS pathogenesis. However, the underlying mechanisms linking salivary gland senescence to the progression and severity of SS remain poorly understood.

In the pathogenesis of SS, immune cell-mediated attacks on exocrine glands represent a central mechanism driving disease progression, ultimately leading to impaired secretory function 17, 18. During the early stages of SS onset and progression, T cell recruitment plays a critical role in driving disease advancement 19-21. By activating bystander immune cells and intensifying chronic inflammation, T cells can further enhance autoimmune responses, thereby promoting later stages of B cell recruitment 22, 23. Recent studies have identified GZMK⁺CD8⁺ T cells as a distinct subset of senescence-associated T cells, which are widely present in aging tissues and exhibit heightened functional activity 24, 25. These GZMK⁺CD8⁺ T cells are characterized by high expression of granzyme K (GZMK), coupled with low levels of granzyme B (GZMB) and interferon-gamma (IFNγ) 24. The central pathogenic mechanism of these T cells is attributed to the production of GZMK, which further promotes the expression of senescence-associated markers and the secretion of senescence-associated secretory phenotype (SASP), thereby contributing to the aging and functional impairment of tissues and organs 26, 27. However, whether GZMK⁺CD8⁺ T cells play a critical role in the progression of SS, and, more specifically, how their production of GZMK contributes to the senescence and secretory dysfunction of acinar cells in SS, remains unclear and requires further investigation.

In this study, we first revealed that acinar cells in the labial glands of SS patients exhibit distinct senescent phenotypes using single-cell RNA sequencing (scRNA-seq), which are closely correlated with salivary secretion dysfunction and SS progression. Furthermore, we identified the recruitment of senescence-associated GZMK⁺CD8⁺ T cells around acinar cells and demonstrated that their production of GZMK may act as a potential risk factor driving acinar cell senescence and impairment in the salivary glands. Mechanistically, we illustrated that GZMK⁺CD8⁺ T cells play a pathogenic role via GZMK production through dual effects. On the one hand, GZMK induced reactive oxygen species (ROS) accumulation and mitochondrial dysfunction, thereby promoting senescent phenotypes in acinar cells from patients with SS; on the other hand, GZMK suppressed TMEM16A, a key chloride (Cl^⁻^) channel protein involved in saliva secretion, inhibiting Cl^⁻^ transport in acinar cells and ultimately impairing saliva production and excretion. Collectively, this study addresses the clinical challenge of understanding why SS exhibits a higher prevalence and more severe symptoms in older individuals, and further elucidates the underlying immunopathological mechanisms of GZMK⁺CD8⁺ T cells through scRNA-seq analysis followed by comprehensive validation, offering new insights into the senescence-associated pathogenesis of SS. Moreover, this study highlights GZMK⁺CD8⁺ T cells as promising therapeutic targets for the precision treatment of SS, particularly in early interventions to alleviate disease progression and restore salivary gland secretory function in patients with SS.

## Materials and methods

### Clinical samples and ethics statement

A total of 4 patients diagnosed with SS and 4 age- and sex-matched healthy controls (HCs) were included for scRNA-seq. Detailed clinical information is provided in **Table S1**. A total of 40 patients diagnosed with SS and 10 age- and sex-matched HCs were included for immunohistochemistry and immunofluorescence. SS patients were selected based on the 2002 American-European Consensus Group (AECG) diagnostic criteria for Sjögren's syndrome 28, 29. Detailed clinical information is provided in **Table S2**. The healthy control group consisted of patients diagnosed with labial mucous cysts without autoimmune diseases or other relevant health conditions. All diagnoses were made at the Department of Oral Medicine, Peking University School and Hospital of Stomatology.

The collection protocol for the human labial gland tissue was reviewed and approved by Peking University School and Hospital of Stomatology Biomedical Ethics Committee, Beijing, China (Approval No. PKUSSIRB-202498061. Date of Approval: 2024-04-22). Written informed consent was obtained from all participants prior to sample collection. Surplus labial gland tissue was obtained at diagnostic biopsy and utilized for sequencing, ensuring no additional harm was inflicted on the participants.

### scRNA-seq and raw data preprocessing

Single-cell preparation, library synthesis, and RNA sequencing were performed by Biomarker Technologies (Beijing, China) according to the company's standard protocols using the 10× Genomics Chromium Single Cell Controller 30. Labial gland biopsy tissues from both SS patients and HCs were processed to generate single-cell suspensions, with cell viability confirmed to exceed 90%. The concentration of the single-cell suspension was approximately 700-1200 cells/μL. The cell aggregation rate was less than 15%, and the cell diameter was maintained below 30 μm. Following cell isolation, RNA was extracted and reverse-transcribed into complementary DNA (cDNA), which was subsequently used for library synthesis. The quality of the libraries was evaluated using the Qubit 4.0 fluorometer and the Agilent 2100 Bioanalyzer. Sequencing was conducted on the Illumina NovaSeq 6000 platform, achieving a minimum sequencing depth of 50,000 reads per cell with 150 bp paired-end reads (PE150). The raw sequencing data were processed using CellRanger version 6.0.0 (10× Genomics), which includes the STAR sequence aligner. The reference genome used for alignment was the human genome GRCh38. Quality control steps were applied to filter out low-quality cells, with the inclusion criteria being a minimum of 500 genes per cell and a mitochondrial gene percentage of less than 20%. Only genes with at least one count in more than three cells were retained for analysis. Gene expression counts were determined by applying unique molecular identifiers (UMIs) to each cell barcode-gene pair. After alignment, cell barcodes were filtered to identify valid cells using the methodology implemented in CellRanger. Only the barcodes corresponding to identified cells were retained for downstream analyses, including clustering, cell type identification, and differential expression analysis.

### Bioinformatic analysis

Bioinformatic analysis was performed using Omicsmart, an interactive online platform for real-time, dynamic data analysis (http://www.omicsmart.com), according to the platform's user guidelines. Briefly, after filtering, 25,961 cells were retained for downstream analysis. The raw count matrix was normalized by total counts per cell and log-transformed. The top 2,000 variable genes were selected, followed by Principal Component Analysis (PCA) and clustering with the Louvain algorithm. Clusters were visualized using Uniform Manifold Approximation and Projection (UMAP). Differentially expressed genes (DEGs) were identified using the Wilcoxon test, with genes showing an average absolute log (Fold Change) greater than 2. Cell type annotations were assigned by integrating DEGs with canonical marker gene expression, supported by literature references. To investigate the functional roles of DEGs, Gene Ontology (GO) and Kyoto Encyclopedia of Genes and Genomes (KEGG) pathway enrichment analyses were performed. Pathways with an adjusted p-value (*p*_adj) < 0.05 were considered significantly enriched. Gene sets for hallmark pathways were retrieved from the Gene Set Enrichment Analysis (GSEA) database. Cell-cell interaction analysis was conducted using CellChat software, based on known receptor-ligand interactions between key cell types and subtypes. Ligand and receptor expression levels were quantified using a threshold derived from the mean log-transformed gene expression across all cell types. Significant interactions were defined as those with a *p*_adj < 0.05 and an average log expression greater than 0.1.

### Immunohistochemistry and immunofluorescence

Immunohistochemistry (IHC) and immunofluorescence (IF) staining were conducted in accordance with standard protocols 31. For IHC, paraffin-embedded tissue sections were deparaffinized and rehydrated through a series of ethanol solutions. Endogenous peroxidase activity was blocked using 3% hydrogen peroxide. Antigen retrieval was performed by heating the sections in citrate buffer. After blocking with 5% bovine serum albumin (BSA), the sections were incubated overnight at 4 ℃ with the primary antibodies. The following day, horseradish peroxidase (HRP)-conjugated secondary antibodies were applied, and antigen staining was developed with DAB solution (G1212, ServiceBio, Wuhan, China). Hematoxylin was used for counterstaining, and the sections were imaged under a light microscope (Nikon, Tokyo, Japan). The histoscore (H-score) was determined by multiplying the percentage of positive cells by the staining-intensity score (0 = no staining; 1 = mild; 2 = moderate; and 3 = strongly positive), resulting in scores ranging from 0 to 300.

For IF staining, tissue sections were first blocked with 5% BSA and then incubated with primary antibodies overnight at 4 ℃. Afterward, sections were incubated with fluorophore-conjugated secondary antibodies (A210, Abbkine, Wuhan, China) for 1 hour at room temperature. Nuclei were stained with DAPI. Images were captured using a fluorescence microscope and scanned with a Panoramic Digital Slide Scanner (3DHISTECH, Budapest, Hungary).

All primary antibodies used are detailed in **Table S3**.

### SMG-C6 cell culture

The rat submandibular gland acinar cell line SMG-C6, gifted by Prof. Guangyan Yu, was cultured under strictly defined conditions according to previously established protocols, including specific medium composition and culture parameters 32-34. Briefly, cells were maintained in a 1:1 mixture of DMEM/F12 medium, supplemented with 2.5% fetal bovine serum (FBS), 5 μg/mL transferrin, 1.1 μMol/L hydrocortisone, 0.1 μMol/L retinoic acid, 2 nMol/L triiodothyronine (T3), 5 μg/mL insulin, 80 ng/mL epidermal growth factor (EGF), 50 μg/mL gentamicin sulfate, 5 mMol/L glutamine, 100 U/mL penicillin, and 100 μg/mL streptomycin. Cells were maintained in a humidified incubator at 37°C with 5% CO₂ to ensure optimal physiological conditions. The culture medium was replenished every 24 hours to maintain nutrient availability and remove metabolic waste. Cells were passaged at 80% confluence using 0.25% trypsin-EDTA, followed by centrifugation and resuspension in fresh medium. All cell culture procedures were performed under aseptic conditions in a biosafety cabinet to prevent contamination. SMG-C6 cells at passages 5-10 were used for all experiments. Cell-line identity was confirmed by rat short tandem repeat (STR) profiling, and mycoplasma contamination was excluded by using the Mycoplasma Detection Kit (HY-K0552, MCE, Shanghai, China).

For co-culture experiments, SMG-C6 cells were co-cultured with GZMK⁺CD8⁺ T cells at a cell ratio of 1:5 using a direct-contact co-culture system for 24 h. For recombinant protein stimulation, SMG-C6 cells were treated with recombinant rat GZMK (rGZMK; CSB-EP010084RA, CUSABIO, Wuhan, China) at a final concentration of 20 ng/mL for 24 h. To assess the dependence of the observed effects on GZMK-associated protease activity, cells were treated with PPACK (HY-122542, MCE, Shanghai, China) at 10 μg/mL for 24 h, either alone or in combination with rGZMK, according to the indicated experimental groups. To investigate the contribution of oxidative stress, SMG-C6 cells were treated with 5 mM N-acetyl-L-cysteine (NAC; MCE, Shanghai, China) or 10 μM MitoTEMPO (MCE, Shanghai, China) for 24 h, either alone or together with rGZMK. Control cells received an equivalent volume of the corresponding vehicle.

### Lentiviral transduction

The *Ano1*-overexpressing lentiviral vector was obtained from TsingKe Biotech (Beijing, China) and used for lentivirus production. HEK293T cells were seeded in 10 cm culture dishes and transfected with the *Ano1*-expressing plasmid, along with packaging plasmids (psPAX2 and pMD2.G), using polyethylenimine (PEI) transfection reagent (HY-W250110E, MCE, Shanghai, China) as the transfection reagent. After 48 hours, the viral supernatants were collected, filtered through a 0.45 μm membrane, and concentrated for downstream use.

SMG-C6 cells were seeded and transduced with the lentiviral particles at a multiplicity of infection (MOI) of 50, supplemented with 8 μg/mL polybrene (HY-112735, MCE, Shanghai, China) to enhance transduction efficiency. After 48 hours, the culture medium was replaced with fresh virus-free complete medium. The lentiviral construct contained a puromycin resistance gene, allowing the selection of successfully transduced cells. 5 μg/mL puromycin (HY-B1743, MCE, Shanghai, China) was added for selection, which was maintained for 72 hours. Transduction efficiency was confirmed by qRT-PCR and Western blot analysis to evaluate *Ano1* overexpression. Successfully transduced cells were expanded and maintained for subsequent experiments.

### Quantitative real-time PCR (qRT-PCR)

Total RNA was extracted from SMG-C6 cells using FastPure Cell/Tissue Total RNA Isolation Kit V2 (RC122, Vazyme, Nanjing, China) following the manufacturer's instructions. RNA concentration and purity were determined by measuring the OD260/OD280 ratio with a NanoDrop 2000 spectrophotometer. One microgram of total RNA was reverse-transcribed into cDNA using ABScript III RT Master Mix (RK20428, ABclonal, Wuhan, China) according to the supplier's protocol. The synthesized cDNA was stored at -20 °C until further use. qRT-PCR was carried out in a 20 μL reaction system using 2X Universal SYBR Green Fast qPCR Mix (RK21203, ABclonal, Wuhan, China). The thermal cycling conditions consisted of an initial denaturation at 95 °C for 3 minutes, followed by 40 cycles of 95 °C for 5 seconds and 60 °C for 30 seconds. β-actin was used as an internal reference gene for normalization, and relative mRNA expression levels were calculated using the 2^⁻ΔΔCt^ method. All primer sequences are provided in **Table S4**.

### Western blot analysis

Total protein was extracted from SMG-C6 cells using RIPA lysis buffer (P0013, Beyotime, Shanghai, China) on ice, followed by ultrasonic disruption and centrifugation at 12,000 × g for 10 min at 4 °C to remove cellular debris. The supernatant was collected, and protein concentrations were determined using a BCA protein assay kit (WB6501, NCM Biotech, Suzhou, China). Equal amounts of protein were separated by sodium dodecyl sulfate-polyacrylamide gel electrophoresis (SDS-PAGE) and transferred onto polyvinylidene fluoride (PVDF) membranes. Membranes were blocked with 5% skim milk in Tris-buffered saline containing 0.1% Tween-20 (TBST) for 1 hour at room temperature, followed by overnight incubation with primary antibodies at 4 °C. After washing three times with TBST, membranes were incubated with HRP-conjugated secondary antibodies at room temperature for 1 hour. Protein signals were visualized using an enhanced chemiluminescence (ECL) reagent (34579, Thermo Fisher Scientific, Waltham, USA), and band intensities were quantified using ImageJ software. Detailed antibody information is provided in **Table S3**.

### Cell cycle analysis

Following the indicated treatments, both floating and adherent SMG-C6 cells were collected to avoid selective loss of damaged or growth-arrested cells. Adherent cells were detached using trypsin without EDTA, combined with the corresponding culture supernatants, and centrifuged at 500 × g for 5 min. The cells were washed twice with ice-cold PBS and resuspended at approximately 1 × 10^6^ cells/mL. For fixation, the cell suspension was slowly added dropwise to ice-cold 70% ethanol with gentle vortexing to minimize cell aggregation. Cells were fixed at -20 °C overnight. After fixation, the cells were centrifuged, washed twice with PBS, and resuspended in staining solution containing propidium iodide (PI; 50 μg/mL) and RNase A (100 μg/mL). Samples were incubated for 30 min at 37 °C in the dark. PI fluorescence was recorded on a linear scale. The percentages of cells in the G0/G1, S, and G2/M phases were calculated from DNA-content histograms using FlowJo software.

### Magnetic separation of CD8^+^ T cells and induction of GZMK⁺CD8⁺ T cells

Rat spleens were aseptically harvested and placed in ice-cold PBS. Single-cell suspensions were prepared by grinding the spleens through a 70-μm cell strainer. After red blood cell lysis with ACK buffer, cells were washed and counted in MACS buffer (PBS containing 0.5% BSA and 2 mM EDTA, pH 7.2). CD8⁺ T cells were purified by positive selection using rat CD8a MicroBeads (130-090-318, Miltenyi Biotec, Germany) according to the manufacturer's instructions. Briefly, up to 1×10⁷ total cells were resuspended in 80 µL of MACS buffer and incubated with 20 µL of CD8a MicroBeads for 15 min at 4 °C. After washing, the cell pellet was resuspended in 500 µL of buffer and loaded onto an LS column placed in the magnetic field of a MACS Separator. The column was washed with 3 mL of buffer, and the magnetically retained CD8⁺ cells were eluted by flushing with 5 mL of buffer after removing the column from the magnetic field. Purified CD8^+^ T cells were cultured in T cell medium supplemented with 25 ng/mL recombinant Rat IL-2 (400-02, PeproTech, USA), 25 ng/mL IL-15 (400-24, PeproTech, USA), and 100 μM STAT5 inhibitor (STAT5-IN-1, HY-101853, MCE, Shanghai, China) for 48 hours.

### Flow cytometry detection of GZMK⁺CD8⁺ T Cells

The purity of the isolated GZMK⁺CD8⁺ T cells was assessed by flow cytometry. Cells were stimulated with phorbol 12-myristate 13-acetate (PMA, 25 ng/mL) and ionomycin (1 µg/mL). Immediately after stimulation, brefeldin A (1 µL/mL) was added to block protein transport and enhance intracellular accumulation of GZMK. Cells were incubated at 37 ℃ in 5% CO₂ for 4 h, then harvested and washed with FACS buffer (PBS containing 2% FBS). Cells were washed once with PBS and stained with a fixable viability dye (BD Horizon Fixable Viability Stain, 1:1000 in PBS) for 15 min at room temperature in the dark. The reaction was quenched with FACS buffer, and cells were centrifuged at 500 × g for 5 min. Surface Fc receptors were blocked with mouse anti-rat CD32 (550271, BD, USA) for 10 min at room temperature, followed by staining with antibodies in FACS buffer for 30 min at 4 °C in the dark. Cells were washed twice with FACS buffer.

Cells were fixed and permeabilized using BD Cytofix Fixation Buffer (554714, BD, USA) according to the manufacturer's instructions. Briefly, 1 × 10^6^ cells were centrifuged at 300 × g for 10 minutes, and the supernatant was carefully removed. The cell pellet was washed twice with cold PBS, resuspended in 250 μL of BD Cytofix Fixation Buffer, and incubated at 4 °C for 30 min. After fixation, cells were washed and resuspended in Perm/Wash Buffer and stored at 4 °C in the dark until further analysis. All antibodies used are detailed in **Table S3**.

### Senescence-associated β-galactosidase (SA-β-Gal) staining

SA-β-Gal activity was assessed using an SA-β-Gal staining kit (C0602, Beyotime, Shanghai, China) following the manufacturer's instructions. Cells in six-well plates were washed once with PBS, and 1 mL of fixation solution was added per well. Cells were fixed at room temperature for 15 minutes, followed by three washes with PBS, 3 minutes each. Next, 1 mL of staining working solution was added to each well, and the plates were sealed with Parafilm. The plates were incubated at 37 °C overnight in a non-CO₂ incubator. After incubation, stained cells were examined and imaged using a light microscope (Nikon, Tokyo, Japan) for further analysis.

Fresh submandibular gland tissues were embedded in optimal cutting temperature compound, rapidly frozen on dry ice and stored at -80 °C. Cryosections of 10 μm thickness were prepared using a cryostat and mounted on positively charged glass slides. Sections were fixed using the fixation solution supplied with the SA-β-Gal staining kit for 15 min at room temperature. After washing with PBS, sections were incubated with freshly prepared SA-β-gal staining solution at pH 6.0 overnight at 37 °C in a non-CO₂ incubator. Sections were subsequently washed, mounted and imaged under a bright-field microscope using identical acquisition settings. For quantification, acinar parenchymal regions were manually delineated as regions of interest. Blood vessels, lymphocytic infiltration foci, and tissue-free spaces were excluded. The blue SA-β-gal-positive area was identified using a predefined color threshold in ImageJ, and the same threshold was applied to all samples.

### Detection of Intracellular and Mitochondrial ROS

Intracellular ROS levels were measured using H_2_DCFDA (HY-D0940, MCE, Shanghai, China), a membrane-permeable fluorescent probe (Ex/Em = 488/525 nm). The assay was performed according to the manufacturer's protocol. A 5 mM stock solution was prepared in anhydrous DMSO and stored at 80 °C in the dark. Before use, the stock solution was diluted in pre-warmed PBS to prepare a 5 μM working solution, which was freshly prepared for each experiment. For staining, cells were harvested and washed twice with PBS for 5 min each. The cell pellet was then resuspended in 1 mL of H_2_DCFDA working solution and incubated at room temperature for 30 min in the dark. After incubation, cells were centrifuged again and the supernatant was carefully removed, and the pellet was washed twice with PBS to eliminate excess dye. Finally, the cell suspension was prepared in 1 mL of PBS, and intracellular ROS levels were analyzed using flow cytometry.

Mitochondrial ROS levels were assessed using MitoSOX^TM^ Red Mitochondrial Superoxide Indicator (M36007, Invitrogen, Waltham, USA), a fluorogenic probe selectively targeting mitochondria (Ex/Em = 396/610 nm). A 5 mM stock solution was freshly prepared before each experiment by dissolving the reagent in 13 μL of anhydrous DMSO. For staining, the stock solution was diluted to a 500 nM working solution in Hank's Balanced Salt Solution (HBSS) supplemented with calcium and magnesium. Cells were incubated with MitoSOX^TM^ Red working solution at 37 °C in a 5% CO₂ incubator for 30 minutes in the dark. Then cells were washed and resuspended in HBSS, and subjected to flow cytometry analysis.

### Transmission electron microscopy

The mitochondrial ultrastructure of SMG-C6 cells, after co-culture with GZMK⁺CD8⁺ T cells or treatment with rGZMK for 24 hours, was examined using transmission electron microscopy (TEM). Briefly, cells were fixed in 2.5% glutaraldehyde at 4 °Covernight, followed by post-fixation in 1% osmium tetroxide (OsO₄) for 1 hour at room temperature. After sequential dehydration in a graded ethanol series, samples were embedded in epoxy resin and sectioned into ultrathin slices using an ultramicrotome. Sections were stained with uranyl acetate and lead citrate to enhance contrast. The prepared grids were examined using a HITACHI HT7800 transmission electron microscope (HITACHI, Tokyo, Japan), and images were captured to assess mitochondrial morphology and structural integrity using the Hitachi TEM system.

### Non-invasive micro-test analysis of Cl^⁻^ transmembrane flux in acinar cells

The real-time transmembrane Cl^⁻^ flux in SMG-C6 cells was measured using non-invasive micro-test technology (NMT) with an NMT Physiolyzer® system (Xuyue, Beijing, China). The direction and rate of Cl^⁻^ flux were recorded to assess Cl^⁻^ transport activity. The assay was conducted following the manufacturer's instructions and established protocols 35-37. Before measurement, the culture medium was replaced with fresh DMEM/F12 medium (pH 7.0), and cells were incubated at 37 °C in a 5% CO₂ incubator for 30 minutes. For Cl^⁻^ flux measurement, the culture dish was transferred to the NMT microscope stage, where cells were selected under a microscope. A Cl^⁻^-selective microelectrode (Xuyue, Beijing, China) was positioned 5 μm from the cell surface, and real-time Cl^⁻^ flux was recorded for 5 minutes per cell. Eight single cells per group were analyzed as biological replicates. Data acquisition and analysis were performed using imFluxes V3.0 software (Xuyue, Beijing, China), with Cl^⁻^ flux rates expressed as pico mol • cm^-2^ • s^-1^. Positive values indicated Cl^⁻^ efflux, whereas negative values represented Cl^⁻^ influx.

### Cl^⁻^ concentration measurement by confocal laser scanning microscopy

Intracellular and extracellular Cl^⁻^ levels in SMG-C6 cells were measured using N-ethoxycarbonylmethyl-6-methoxyquinolinium bromide (MQAE, S1082, Beyotime, Shanghai, China), a chloride-sensitive fluorescent probe (Ex/Em = 355/460 nm). As Cl^⁻^ concentration increases, MQAE fluorescence intensity decreases proportionally. For MQAE staining, cells were incubated in Krebs-HEPES buffer containing 5 mM MQAE at a density of 1 × 10^7^ cells/mL. Following incubation for 30 minutes, cells were washed with Krebs-HEPES buffer to remove excess probe. To visualize cell membranes, DiI (C1036, Beyotime, Shanghai, China) was used for counterstaining (Ex/Em = 549/565 nm). Confocal laser scanning microscopy (CLSM) was performed using a Nikon AX microscope (Nikon, Tokyo, Japan), and images were captured using NIS-Elements Viewer software (Nikon, Tokyo, Japan).

### Mice and PPACK treatment

Female NOD/ShiLtJ mice were obtained from GemPharmatech (Nanjing, China), and maintained under specific pathogen-free conditions at controlled temperature and humidity with a 12-h light/dark cycle. All animal procedures were approved by the Institutional Animal Care and Use Committee of Peking University Health Science Center under approval number DLASBD0428 and were performed in accordance with institutional guidelines for the care and use of laboratory animals.

At 8 weeks of age, NOD/ShiLtJ mice were randomly assigned to the PBS and PPACK treatment groups with 6 mice per group. PPACK was dissolved in sterile PBS and administered intraperitoneally at 2 mg/kg every other day for two weeks. Control mice received an equivalent volume of PBS via the same route and schedule. Body weight, blood glucose and stimulated saliva were monitored biweekly throughout the intervention period. Peripheral blood was collected at the endpoint. The submandibular glands were subsequently dissected. One gland was processed immediately for flow cytometry, whereas the contralateral gland was divided into anatomically matched portions for fresh-frozen SA-β-gal staining and formalin-fixed paraffin-embedded histological analyses.

### Measurement of stimulated salivary flow rate

Mice were anesthetized with 1.25% tribromoethanol at 15 μL/g body weight. Pilocarpine hydrochloride (5 mg/kg; HY-B0726, MCE, Shanghai, China) was then administered intraperitoneally to stimulate salivation. Five minutes after pilocarpine administration, saliva was continuously collected from the oral cavity for 15 min. Salivary flow rate was normalized to collection time and body weight and calculated. Saliva collection was performed at the same time of day and under identical experimental conditions to minimize circadian and procedural variability.

### Measurement of anti-SSA and anti-SSB autoantibodies

Peripheral blood was collected at the experimental endpoint by retro-orbital bleeding under anesthesia. Blood samples were allowed to clot at room temperature for 30 min and centrifuged at 2,000 × g for 10 min at 4 °C. The serum fraction was collected and stored at -80 °C until analysis. Serum anti-SSA and anti-SSB autoantibody levels were measured using commercially available mouse anti-SSA and anti-SSB ELISA kits (ELK9993, ELK7184, ELK Biotechnology, Wuhan, China) according to the manufacturer's instructions. Standards and samples were added to the antibody-coated plates and incubated for the specified duration, followed by sequential incubation with the detection antibody and HRP-conjugated reagent. After addition of tetramethylbenzidine substrate, the reaction was terminated using stop solution, and absorbance was measured at 450 nm using a microplate reader. Autoantibody concentrations were calculated from the corresponding standard curves and expressed as ng/mL.

### Statistical analysis

All experimental data are presented as the mean ± SD based on at least three independent experiments. Statistical analyses were performed using IBM SPSS Statistics for Windows, version 26.0 (IBM Corp., Armonk, USA). Data normality was assessed using the Kolmogorov-Smirnov test. An unpaired two-tailed Student's *t*-test was used to compare the two groups. For comparisons involving multiple groups, one-way analysis of variance (ANOVA) followed by the Tukey's multiple-comparison test was applied if the data followed a normal distribution and homogeneity of variances. When these criteria were not met, the Dunnett T3 test was employed. To evaluate the association between IHC scores and clinicopathological parameters in SS patients, Spearman's rank correlation analysis was performed. Statistical significance was defined as a *p*-value less than 0.05.

## Results

### Single-cell transcriptomics revealed key features of acinar cell senescence and secretory dysfunction in SS labial glands

Labial gland biopsy samples were collected from four SS patients and four age- and sex-matched HCs for scRNA-seq using the 10× Genomics platform **(Fig. 1A)**. After stringent quality control (QC) filtering, a total of 25,961 cells were retained for downstream analysis, including dimensionality reduction, clustering, and annotation. The QC overview of the filtered cells is provided in Fig. S1. Based on the differential expression of cluster-specific genes and known marker genes, these cells were categorized into 10 major cell types **(Fig. 1B-E)**. Given the functional diversity of epithelial cells in labial glands, the epithelial cell population was further subdivided into two distinct groups, acinar cells and ductal cells, based on their specific marker genes **(Fig. 1F-H)**. Proportional analysis revealed a significant reduction in the total epithelial cell population in SS patients compared to HCs, and notably, acinar cells showed a more pronounced reduction than ductal cells, suggesting that acinar cells are primarily damaged during SS progression and play a critical role in the salivary gland dysfunction of SS patients.

To further elucidate the senescence characteristics of labial gland tissue in SS patients, we assessed senescence-related gene sets in each type of resident tissue cells, including epithelial cells, myoepithelial cells, endothelial cells, fibroblasts, smooth muscle cells (SMCs), and melanocytes. Senescence-related pathway gene sets were derived from previously published studies (**Table S5**) 38-45, and senescence scores were calculated for these cells in SS and HC groups. Overall, the senescence scores in the SS group were significantly higher than those in the HC group **(Fig. 1I)**. Specifically, among all the resident tissue cell types, epithelial cells exhibited the highest senescence scores in SS patients **(Fig. 1J)**, which were significantly elevated compared to HCs **(Fig. 1K)**. Additionally, acinar cells displayed higher senescence scores than ductal cells **(Fig. 1L)**. Further analysis of senescence markers revealed that the acinar cells from SS patients exhibited significantly increased gene expression of SA-β-Gal, p16^INK4a^, p21, and the SASP factor IL-6 compared to HCs **(Fig. 1M)**. These findings underscore the prominent senescent phenotypes of acinar cells in SS.

For the evaluation of secretory dysfunction in SS epithelial cells, we compiled and analyzed gene sets related to salivary secretion, including ion channel and water channel proteins, from previous studies (**Table S6**) 36, 46-54. Secretory function scores were calculated for epithelial cells in both SS and HC groups, with the SS group showing significantly lower scores compared to HCs, indicating a marked impairment of salivary secretion **(Fig. 1N)**. Moreover, the results revealed that acinar cells in the SS group had significantly lower secretory function scores than ductal cells, suggesting a more severe impairment in acinar cells **(Fig. 1O)**. In summary, the above scRNA-seq results demonstrated that acinar cells in SS patients exhibit distinct senescent phenotypes and secretory dysfunction.

### Histological validation confirms labial gland senescence and its correlation with salivary dysfunction and disease progression in SS patients

Based on the single-cell transcriptomics findings, we further validated the expression of key senescence markers, including SA-β-Gal, p16^INK4a^, p21, and the SASP factor IL-6, in labial gland biopsy tissue sections from SS patients and HCs using IHC. Moreover, we performed Spearman's rank correlation analysis to assess the relationship between the histoscores of senescence markers and several clinicopathological indicators reflecting SS progression and severity, including unstimulated whole saliva flow rate, patient age, labial gland lymphocytic focus scores, as well as serum anti-SSA levels.

IHC analysis revealed that SS labial glands exhibited high expression levels of senescence markers SA-β-Gal, p16^INK4a^, p21, and IL-6 **(Fig. 2A)**. Quantitative analysis of the histoscores confirmed that the expression levels of these markers were significantly higher in SS patients compared to HCs, further supporting the findings from single-cell transcriptomics** (Fig. 2B)**.

Spearman's rank correlation analysis showed that the histoscores of SA-β-Gal, p16^INK4a^, p21, and IL-6 were negatively correlated with the unstimulated whole saliva flow rate in SS patients, indicating a close association between the senescent phenotypes in labial glands and the impairment of salivary secretion **(Fig. 2C)**. Moreover, these senescence markers were positively correlated with other indicators of SS progression, including patient age, lymphocytic focus scores, and serum anti-SSA/SSB levels **(Fig. 2D-F)**. These findings suggest that labial gland senescence plays a critical role in the progression of SS and may contribute to the exacerbation of its clinical manifestations, particularly the dysfunction in salivary secretion.

### Senescence-associated GZMK⁺CD8⁺ T cells are highly enriched and functionally active in SS labial glands, exhibiting significant interactions with acinar cells

In the pathogenesis of SS, immune cell-mediated attacks on acinar cells represent a central driver of disease onset and progression. In particular, T cell recruitment plays a critical role in the early stages of SS. Therefore, to identify which specific T cell subsets may drive acinar cell senescence and secretory dysfunction, we performed a detailed re-annotation and subgroup analysis of labial gland T cell populations. We also divided SS patients and HCs into younger (< 65 years old) and older (≥ 65 years old) cohorts, providing clearer insights into the role of senescence-associated T cells in SS progression.

Single-cell sequencing results revealed that the T/NK cell population was re-annotated and subclassified into 14 distinct subsets **(Fig. 3A-C)**. Proportional analysis of these subsets in the elderly SS, young SS, and age- and sex-matched HC groups identified a specific senescence-associated T cell subset: GZMK⁺CD8⁺ T cells, which were also highly associated with SS **(Fig. 3D)**. In detail, this subset was least abundant in the young HC group, increased in the elderly HC group, and exhibited markedly elevated proportions in both the young and elderly SS groups, with the highest abundance observed in the elderly SS group. These findings suggest a potential association between the recruitment of senescence-associated GZMK⁺CD8⁺ T cells in salivary glands and SS progression.

To further investigate the functional activity of GZMK⁺CD8⁺ T cells, DEG analysis was performed on this subset **(Fig. 3E)**. A significantly higher number of upregulated functional genes were detected in GZMK⁺CD8⁺ T cells from the SS group compared to the HC group, indicating their heightened functional activity in SS patients. Since GZMK⁺CD8⁺ T cells exert their effects primarily through the production of GZMK, the expression levels of the key functional gene *GZMK* were further analyzed across all groups **(Fig. 3F)**. The results showed that *GZMK* expression was significantly elevated in both the young and elderly SS groups compared to the young and elderly HC groups, with the highest expression observed in the elderly SS group.

Subsequently, CellChat analysis was performed to explore the intercellular communication between GZMK⁺CD8⁺ T cells and various resident tissue cell types **(Fig. 3G)**. Significant interactions were observed between GZMK⁺CD8⁺ T cells and acinar cells, with a higher number of ligand-receptor pairs compared to other cell types. The top 15 ligand-receptor pairs involved in the interaction between GZMK⁺CD8⁺ T cells and acinar cells are shown in **Fig. 3H**. These findings further support the potential critical role of GZMK⁺CD8⁺ T cells in inducing acinar cell damage in SS.

### GZMK⁺CD8⁺ T cells produce high levels of GZMK around SS acinar cells, correlating with the salivary dysfunction and disease severity of SS patients

To further confirm the scRNA-seq findings of GZMK⁺CD8⁺ T cell infiltration and elucidate the correlation between GZMK production and SS severity, histological analysis was performed using labial gland biopsy tissue sections from SS patients and HCs. The infiltration of GZMK⁺CD8⁺ T cells and the expression levels of GZMK in labial glands were assessed via IF and IHC staining. Spearman's rank correlation analysis was then conducted between the histoscores of GZMK and several clinicopathological indicators reflecting SS progression and severity, including unstimulated whole saliva flow rate, duration of xerostomia, labial gland lymphocytic focus scores, and serum anti-SSA/SSB levels.

IF results showed that GZMK⁺CD8⁺ T cells infiltrated the regions around acinar cells in the labial glands of both elderly and young SS patients, with a higher level of infiltration in the elderly SS group, and both groups exhibited higher infiltration levels than HCs **(Fig. 4A)**. IHC analysis further confirmed the expression of GZMK in the labial glands of SS patients **(Fig. 4B-C)**. The results showed that the expression levels of GZMK were significantly higher in both the elderly and young SS groups compared to the HC group, with the elderly SS group showing the highest GZMK histoscore.

Moreover, Spearman's rank correlation analysis revealed that the histoscores of GZMK were negatively correlated with the unstimulated whole saliva flow rate in SS patients, suggesting that higher GZMK expression is associated with more severe salivary dysfunction **(Fig. 4D)**. In addition, GZMK histoscores were positively correlated with the duration of xerostomia, lymphocytic focus scores, and serum anti-SSA/SSB levels **(Fig. 4E-G)**. To determine whether GZMK expression was associated with disease severity independently of relevant clinical factors, we performed multivariable linear regression analyses. Using histological focus score of labial glands as the dependent variable, sex, age, serum anti-SSA/SSB antibody scores, duration of xerostomia, and GZMK H-score were simultaneously entered as covariates **(Table S7)**. Serum anti-SSA/SSB antibody scores were independently associated with a higher focus score (standardized β = 0.483, *P* = 0.003). Importantly, GZMK H-scores also remained positively associated with focus score after adjustment for the other covariates (standardized β = 0.394, *P* = 0.03). Sex, age, and duration of xerostomia were not independently associated with focus score. No substantial multicollinearity was detected among the included variables, with all variance inflation factors below 2.

These results indicate a close link between GZMK levels in salivary glands and the severity of SS **(Fig. 4H)**. The mechanisms through which GZMK drives acinar cell senescence and secretory dysfunction will be further explored in the following sections of this study.

### GZMK⁺CD8⁺ T cells induce increased ROS accumulation and mitochondrial dysfunction, leading to senescent phenotypes in acinar cells

To determine whether GZMK mediated the effects of GZMK⁺CD8⁺ T cells on salivary acinar cells, SMG-C6 cells were either co-cultured with GZMK⁺CD8⁺ T cells or exposed to rGZMK, with or without PPACK. Both GZMK⁺CD8⁺ T cell co-culture and rGZMK treatment markedly increased SA-β-gal staining compared with untreated control cells, whereas PPACK significantly reduced the SA-β-gal-positive area in both experimental settings **(Fig. 5A)**. Consistently, transcriptional analysis showed increased expression of multiple inflammatory and senescence-associated genes, including *Tnf, Il1a, Il1b, Il6, Ccl2, Ccl5, Mmp9, Cdkn2a,* and* Cdkn1a*, following co-culture or rGZMK stimulation; these changes were attenuated by PPACK treatment **(Fig. 5B)**. Western blotting further showed increased p16^INK4a^ protein abundance in co-cultured and rGZMK-treated cells, with lower expression following PPACK treatment **(Fig. 5C)**. Cell-cycle analysis demonstrated an increased proportion of cells in the G0/G1 phase and a corresponding reduction in the S-phase fraction after GZMK⁺CD8⁺ T cell co-culture or rGZMK treatment. PPACK partially normalized the cell-cycle distribution **(Fig. 5D)**. Together, these findings indicate that extracellular GZMK contributes to the induction of a senescence-associated phenotype in SMG-C6 cells.

To investigate the mechanisms through which GZMK⁺CD8⁺ T cells induce senescent phenotypes in acinar cells, we performed GSEA and KEGG analyses on the scRNA-seq data. GSEA analysis of hallmark pathways indicated a pronounced activation of the ROS pathway in SS acinar cells **(Fig. 5E)**. KEGG analysis revealed significant enrichment and downregulation of the oxidative phosphorylation pathway in SS acinar cells, with 28 DEGs downregulated and only 13 upregulated, suggesting potential mitochondrial dysfunction in the cells **(Fig. S2A-B)**. These findings suggest that SS acinar cells may exhibit high levels of ROS accumulation and mitochondrial dysfunction, which could contribute to the expression of senescent phenotypes.

To further validate whether GZMK can induce ROS accumulation and mitochondrial dysfunction in acinar cells, we conducted *in vitro* experiments using SMG-C6 cells. Direct measurements showed that both co-culture and rGZMK treatment significantly increased intracellular ROS and mitoROS levels. PPACK markedly attenuated these increases **(Fig. 5F, Fig. S2C)**. Transmission electron microscopy (TEM) further revealed mitochondrial swelling, disrupted cristae, and vacuole-like structural changes in co-cultured and rGZMK-treated cells. These ultrastructural abnormalities were less pronounced after PPACK treatment **(Fig. 5G)**.

To further assess whether oxidative stress contributed to the rGZMK-induced phenotype, SMG-C6 cells were treated with the general antioxidant NAC or the mitochondria-targeted antioxidant MitoTEMPO. Both treatments significantly reduced the rGZMK-induced increase in SA-β-gal-positive area **(Fig. 5H)**. NAC and MitoTEMPO also partially restored cell-cycle progression, as indicated by a reduction in G0/G1 accumulation and an increase in the S-phase fraction compared with rGZMK treatment alone **(Fig. 5I)**. In parallel, both antioxidants markedly decreased intracellular ROS and mitoROS levels and alleviated rGZMK-induced mitochondrial ultrastructural damage **(Fig. 5J, K)**.

Collectively, these results support a model in which extracellular GZMK promotes oxidative stress and mitochondrial dysfunction, thereby inducing senescence-associated phenotypes in salivary acinar cells.

### GZMK suppresses Cl^⁻^ channel TMEM16A expression, thereby inhibiting Cl^⁻^ transport in acinar cells

To better understand how GZMK contributes to acinar cell dysfunction, we analyzed scRNA-seq data to examine the expression of secretion-related channel proteins in SS acinar cells. We identified TMEM16A, a Cl^⁻^ channel protein, as the most significantly downregulated in SS acinar cells, with its gene expression (*ANO1*) significantly lower in SS patients compared to HCs, and the lowest levels observed in the elderly SS group **(Fig. 6A-B)**. Subsequently, we validated these findings using IHC. The results showed that the expression of TMEM16A in labial gland tissue was significantly lower in both elderly and young SS patients compared to HCs, with the lowest histoscores observed in the elderly SS group **(Fig. 6C-D)**.

To further investigate whether GZMK can inhibit the expression of TMEM16A in acinar cells, we performed *in vitro* experiments using SMG-C6 cells. The cells were treated with rGZMK, and TMEM16A expression at both the mRNA and protein levels was assessed using qRT-PCR and Western blot **(Fig. 6E-G)**. The results revealed significant downregulation of both TMEM16A gene and protein levels in rGZMK-treated SMG-C6 cells, confirming that GZMK suppresses the expression of TMEM16A in acinar cells.

Given that TMEM16A is essential for Cl^⁻^ transport and electrolyte balance in acinar cells, we further assessed whether GZMK can inhibit the Cl^⁻^ transport efficacy of TMEM16A. First, we employed the Non-invasive Micro-test System (NMT), a technique allowing precise measurement of ion transport across cell membranes, to measure the Cl^⁻^ flux in acinar cells in real time **(Fig. 6H-J,** Video S1**)**. The results revealed a significant reduction in the Cl^⁻^ efflux rate in rGZMK-treated SMG-C6 cells, which decreased to about one-third of that observed in control cells. In contrast, when TMEM16A was overexpressed in SMG-C6 cells, the Cl^⁻^ efflux rate significantly recovered, reaching levels twice those of the rGZMK-treated group. The overexpression of TMEM16A in SMG-C6 cells was validated by qRT-PCR and Western blot analysis **(Fig. S3)**.

Additionally, the intracellular Cl^⁻^ concentrations in SMG-C6 cells before and after rGZMK treatment were evaluated using CLSM **(Fig. 6K-L).** The Cl^⁻^ ions were labeled with the N-(Ethoxycarbonylmethyl)-6-methoxyquinolinium bromide (MQAE) probe, and the cell membrane was stained with DiI. The intracellular MQAE fluorescence intensity was then measured. After rGZMK treatment, the mean fluorescence intensity (MFI) of MQAE in the cytoplasm of SMG-C6 cells decreased significantly compared to the control group, indicating impaired Cl^⁻^ efflux. In the TMEM16A overexpression group, the intracellular MQAE MFI recovered to levels similar to those in the vector group, suggesting restoration of Cl^⁻^ efflux capability.

To determine whether the impairment of chloride transport induced by extracellular GZMK was dependent on its associated serine protease activity, SMG-C6 cells were treated with rGZMK in the presence or absence of PPACK. NMT showed that rGZMK markedly reduced the Cl⁻ efflux rate compared with untreated control cells. PPACK alone did not noticeably alter basal Cl⁻ efflux, whereas the addition of PPACK significantly increased the Cl⁻ efflux rate in rGZMK-treated cells compared with rGZMK treatment alone **(Fig. S4A)**. These findings were independently supported by MQAE fluorescence imaging. Quantitative analysis showed that rGZMK significantly decreased the relative MQAE fluorescence intensity compared with the control group **(Fig. S4B, C)**. PPACK significantly attenuated the rGZMK-induced reduction in MQAE fluorescence, consistent with restoration of chloride transport. Together, these results provide pharmacological evidence that rGZMK-induced chloride transport dysfunction depends on GZMK-associated serine protease activity.

In summary, these findings suggest that GZMK inhibits the expression of Cl^⁻^ channel TMEM16A on acinar cells as well as its Cl^⁻^ transport function, thereby impairing salivary secretion in SS.

### Inhibition of GZMK with PPACK ameliorates salivary gland dysfunction and validates the GZMK-associated pathogenic mechanism in NOD mice

To determine the effect of GZMK in salivary gland dysfunction* in vivo*, age-matched NOD mice were treated with PBS or PPACK, a reported inhibitor of GZMK-associated serine protease activity. PPACK was given via intraperitoneal injection at a dose of 2 mg/kg every other day for 2 consecutive weeks, starting at 8 weeks of age. PPACK treatment significantly increased the stimulated salivary flow rate compared with PBS treatment, indicating an improvement in salivary secretory function **(Fig. 7A)**. Serum levels of anti-SSA and anti-SSB antibodies were significantly reduced in NOD mice with PPACK treatment **(Fig. 7B, C)**. Histopathological examination of the submandibular glands showed prominent lymphocytic infiltration in PBS-treated NOD mice, whereas the size of inflammatory foci was markedly reduced following PPACK treatment **(Fig. 7D)**. These results indicate that pharmacological inhibition of GZMK-associated protease activity alleviates glandular inflammation and secretory dysfunction in NOD mice.

We next investigated whether PPACK treatment affected the glandular GZMK⁺CD8⁺ T cell population. Flow-cytometric analysis of the salivary gland showed that the frequency of GZMK⁺ CD8⁺ T cells was significantly lower in PPACK-treated mice than in PBS-treated controls **(Fig. 7E)**. This finding suggests that PPACK treatment reduces the accumulation of GZMK-expressing CD8⁺ T cells within the diseased salivary gland microenvironment. To further assess senescence-associated changes *in vivo*, SA-β-gal staining was performed on submandibular gland sections from PBS and PPACK-treated NOD mice.

Compared with the PBS group, submandibular gland tissues from PPACK treated mice exhibited markedly reduced blue SA-β-gal staining within the acinar parenchyma. Quantitative analysis confirmed a significant decrease in the percentage of SA-β-gal-positive area following PPACK treatment **(Fig. 7F)**. Immunohistochemical staining demonstrated that p16^INK4a^ expression **(Fig. 7G)** in submandibular gland tissues was significantly decreased and TMEM16A expression **(Fig. 7H)** was increased following PPACK administration. These findings are consistent with the *in vitro* observations and support a model in which GZMK contributes to senescence-associated glandular injury and TMEM16A suppression in Sjögren's syndrome.

The systemic tolerability of PPACK was also evaluated. Body weight and blood glucose remained stable throughout the treatment period, with no significant difference between the two groups **(Fig. S5A, B).** In addition, no obvious histopathological abnormalities were observed in the liver, kidney, or lung tissues of PPACK-treated mice compared with PBS-treated controls **(Fig. S5C)**. These observations suggest that PPACK was well tolerated under the dose and treatment schedule used in this study.

Collectively, these *in vivo* data demonstrate that pharmacological inhibition of GZMK-associated protease activity improves salivary secretion, reduces glandular lymphocytic infiltration and GZMK⁺CD8⁺ T cell abundance, attenuates senescence-associated changes, and restores TMEM16A expression in NOD mice. These findings provide* in vivo* support for the pathogenic relevance of GZMK-centered mechanism identified in salivary gland epithelial cells.

## Discussion

Sjögren's syndrome (SS) is a representative autoimmune disease that primarily affects the salivary glands and other exocrine glands, predominantly impacting middle-aged and elderly women, with typical onset around the age of 55 3, 5, 12. Elderly SS patients are more susceptible to multi-organ involvement, with higher disease activity and severity, particularly in the joints, lungs, and peripheral nervous system 13, 15. More concerningly, these patients also have an elevated risk of lymphoma, which significantly worsens their prognosis 8, 9. Several clinical studies have provided evidence highlighting the relationship between aging and SS severity. In a study of 21 patients with SS whose disease began after age 65, 71.4% reported dry mouth symptoms, and 76.1% exhibited dry eye symptoms 15.

While the clinical association between aging and SS is well recognized, the underlying mechanisms linking senescence-related changes to the progression of SS remain insufficiently understood. Among the few studies that have explored the senescence-related mechanisms in SS, one foundational study reported reduced salivary gland stem cell (SGSC) numbers, impaired differentiation, and deficient regeneration 55. Moreover, the study showed that the presence of SS-associated proinflammatory cytokines, including IL-6, IFN-α, and TNF, exacerbates the senescent state of SGSCs, further impeding their differentiation into acinar cells, which is a critical factor in glandular dysfunction 55. Subsequent research has explored the senescence of salivary gland progenitor cells (SGPCs) in SS pathophysiology, as these cells are essential for maintaining glandular homeostasis 56. This study demonstrated a significant increase in p16^+^ senescent cells within both the overall salivary gland epithelium and the basal striated duct (BSD) niche in primary SS patients 56. These findings highlight the close link between premature SGPC senescence and salivary gland dysfunction, as well as localized disease pathology in SS.

Despite these advances, current research remains limited by a lack of high-resolution, comprehensive analyses of acinar cell senescence and its impact on SS progression at the single-cell level. Therefore, our study addressed this issue by employing scRNA-seq to clarify the key phenotypes of acinar cell senescence, providing new evidence on the complex relationship between acinar cell senescence and SS progression. Using scRNA-seq and histological validation, we identified that acinar cells in SS patients exhibit increased expression of SA-β-Gal, p16^INK4a^, and p21, as well as the SASP factor IL-6, which were all correlated with critical clinicopathological indicators of SS patients, including patient age, unstimulated whole saliva flow rate, lymphocytic focus scores in labial glands, and serum anti-SSA/SSB levels. Cellular senescence is characterized by several defining features, such as the activation of SA-β-gal and the upregulation of critical cell cycle inhibitors, including p16^INK4a^ and p21 57. Another key hallmark of this process is the secretion of SASP, which comprises proinflammatory cytokines, chemokines, extracellular matrix proteins, and other bioactive molecules 58. Our study provides new insights into the role of acinar cell senescence in SS progression, highlighting their correlation with disease severity.

Regarding the mechanisms that induce the senescent phenotype, the accumulation of intracellular ROS and mitochondrial dysfunction are particularly critical 59-62. ROS induces oxidative damage to mitochondrial DNA and proteins, leading to compromised mitochondrial function, which in turn exacerbates ROS production, creating a self-reinforcing cycle that perpetuates senescence 63-66. Mitochondrial dysfunction further impacts adenosine triphosphate (ATP) production by disrupting the oxidative phosphorylation pathways, thus promoting the expression of senescence markers and the production of SASP 65-68. In this study, we identified GZMK-induced ROS accumulation and mitochondrial dysfunction in acinar cells as a key factor contributing to salivary gland senescence and dysfunction in SS, suggesting that amelioration of mitochondrial dysfunction may serve as a promising therapeutic strategy. Several studies have also shown that oxidative stress and mitochondrial dysfunction are key drivers of other autoimmune diseases 69. For instance, in rheumatoid arthritis (RA), the overexpression of glucose-6-phosphate dehydrogenase, which enhances oxidative stress resistance, has been linked to disease exacerbations 70. Similarly, mitochondrial oxidative stress has been identified as a significant driving factor in systemic lupus erythematosus (SLE), with antioxidant therapies such as acetylcysteine showing promise in treatment 71, 72. Therefore, understanding the relationship between oxidative stress and the initiation and persistence of autoimmune conditions is essential.

GZMK⁺CD8⁺ T cells were first identified in tissues of aged mice through scRNA-seq, and were thus defined as a specific subset of senescence-associated T cells 24. Additionally, peripheral blood mononuclear cells (PBMCs) from elderly human subjects also showed a high prevalence of GZMK⁺CD8⁺ T cells 24. These senescence-associated T cells are characterized by high expression of GZMK, low expression of GZMB and IFN-γ, and co-expression of the transcription factor Tox and immune checkpoint molecule PD-1 24. They also play a critical role in the pathogenesis of various autoimmune diseases 26. In RA, they are enriched in synovial fluid and tissue, where they stimulate fibroblasts to release inflammatory cytokines via GZMK, driving chronic inflammation and joint damage 73, 74. In lupus nephritis, GZMK⁺CD8⁺ T cells contribute to disease progression through clonal expansion and cytotoxic, inflammatory effects 75. In chronic graft-versus-host disease (cGVHD) and its associated bronchiolitis obliterans syndrome (BOS), these cells undergo clonal expansion and infiltration, promoting fibrosis and lung dysfunction by inducing fibrotic markers such as collagen and fibronectin 76. Elevated IL-15 levels in cGVHD patients can further enhance the differentiation of CD8^+^ T cells into GZMK⁺CXCR6⁺ T cells, exacerbating the tissue damage 76. Another study confirmed that elevated IL-15 levels can promote the differentiation of CD8^+^ T cells into GZMK⁺CXCR6⁺CD8⁺ T cells 25. Our study further clarifies the intricate interaction between GZMK⁺CD8⁺ T cells and SS acinar cells, revealing the mechanism by which these cells induce senescent phenotypes and impair the Cl^⁻^ channel protein TMEM16A, emphasizing their specific role in the progression of SS.

Acinar cell secretory dysfunction, the primary cause of xerostomia in SS patients, is largely determined by the expression and functionality of multiple secretion-related channel proteins on their surface 77, 78. In healthy salivary glands, the secretory function of acinar cells is finely regulated by multiple ion and water channel proteins, such as the Ca²⁺-activated K⁺ channel MaxiK, the Ca²⁺-dependent Cl^⁻^ channel protein TMEM16A, the Na⁺/K⁺/2 Cl^⁻^ cotransporter NKCC1, the Na⁺/H⁺ exchanger NHE1, and the water channel AQP5, which are critical for maintaining salivary production and secretion 79, 80. TMEM16A, predominantly located on the apical side of acinar cells, plays a vital role in facilitating Cl^⁻^ efflux, maintaining electrolyte balance and water transport, and ultimately ensuring normal salivary secretion 81-83. Previous studies have shown that silencing TMEM16A in the submandibular glands of mice via siRNA significantly reduces the salivary flow 37, 84. Previous studies have reported context-dependent crosstalk between TMEM16A and mitochondrial ROS, with some models showing that TMEM16A regulates mitochondrial dynamics, while others treat them as parallel pathways 85, 86. However, scavenging ROS with NAC or MitoTEMPO failed to rescue GZMK-induced TMEM16A downregulation in salivary acinar cells, indicating that TMEM16A suppression is not secondary to ROS accumulation under these conditions. Collectively, our data support a bifurcated model in which GZMK drives a mitochondrial dysfunction associated senescence program while independently suppressing TMEM16A-mediated chloride transport. Importantly, the *in vivo* PPACK intervention extended our *in vitro* findings to an SS-relevant disease model. PPACK treatment improved stimulated salivary secretion and reduced lymphocytic infiltration in the submandibular glands of NOD mice. The findings are consistent with the proposed role of GZMK in promoting senescence-associated glandular injury and impairing TMEM16A-related secretory function.

Several limitations of this study should be acknowledged. The discovery phase of our single-cell RNA sequencing was performed on a relatively small cohort comprising four SS patients and four healthy controls. Although this sample size is comparable to many exploratory scRNA-seq studies, we recognize that it limits the statistical power to draw definitive conclusions regarding the absolute prevalence or global population frequencies of the identified cell subsets. Therefore, we have used cautious, descriptive language when presenting proportional differences derived from this discovery set. Importantly, the key molecular signatures and functional phenotypes identified herein were further validated in larger independent cohorts. Nevertheless, future studies with expanded, multicenter patient cohorts are warranted to comprehensively establish the clinical and pathological significance of the GZMK⁺CD8⁺ T cell subset in SS. Furthermore, PPACK is not exclusively selective for GZMK and may inhibit other serine proteases. The *in vivo* findings should be interpreted as pharmacological support for the GZMK-associated mechanism rather than definitive evidence of target-exclusive inhibition. Future studies using genetic GZMK deletion or highly selective neutralization strategies will be required to further establish target specificity.

## Conclusions

This study offers new insights into the mechanisms by which GZMK⁺CD8⁺ T cells induce acinar cell senescence and secretory dysfunction in SS. By integrating single-cell sequencing and histological validation, we demonstrated that acinar cells in SS patients exhibit pronounced senescent phenotypes and impaired secretion, which closely correlate with SS progression and severity. Mechanistically, GZMK induces ROS accumulation and mitochondrial dysfunction, thereby inducing senescence-associated phenotypes in acinar cells. Moreover, GZMK reduces the expression of Cl^⁻^ channel protein TMEM16A and impairs Cl⁻ transport, ultimately compromising salivary secretion and exacerbating glandular dysfunction. These findings point to GZMK⁺CD8⁺ T cells and related pathways as promising therapeutic nodes for early intervention in SS.

## Supplementary Material

Supplementary figures and tables.

Supplementary video 1,

## Figures and Tables

**Figure 1:**
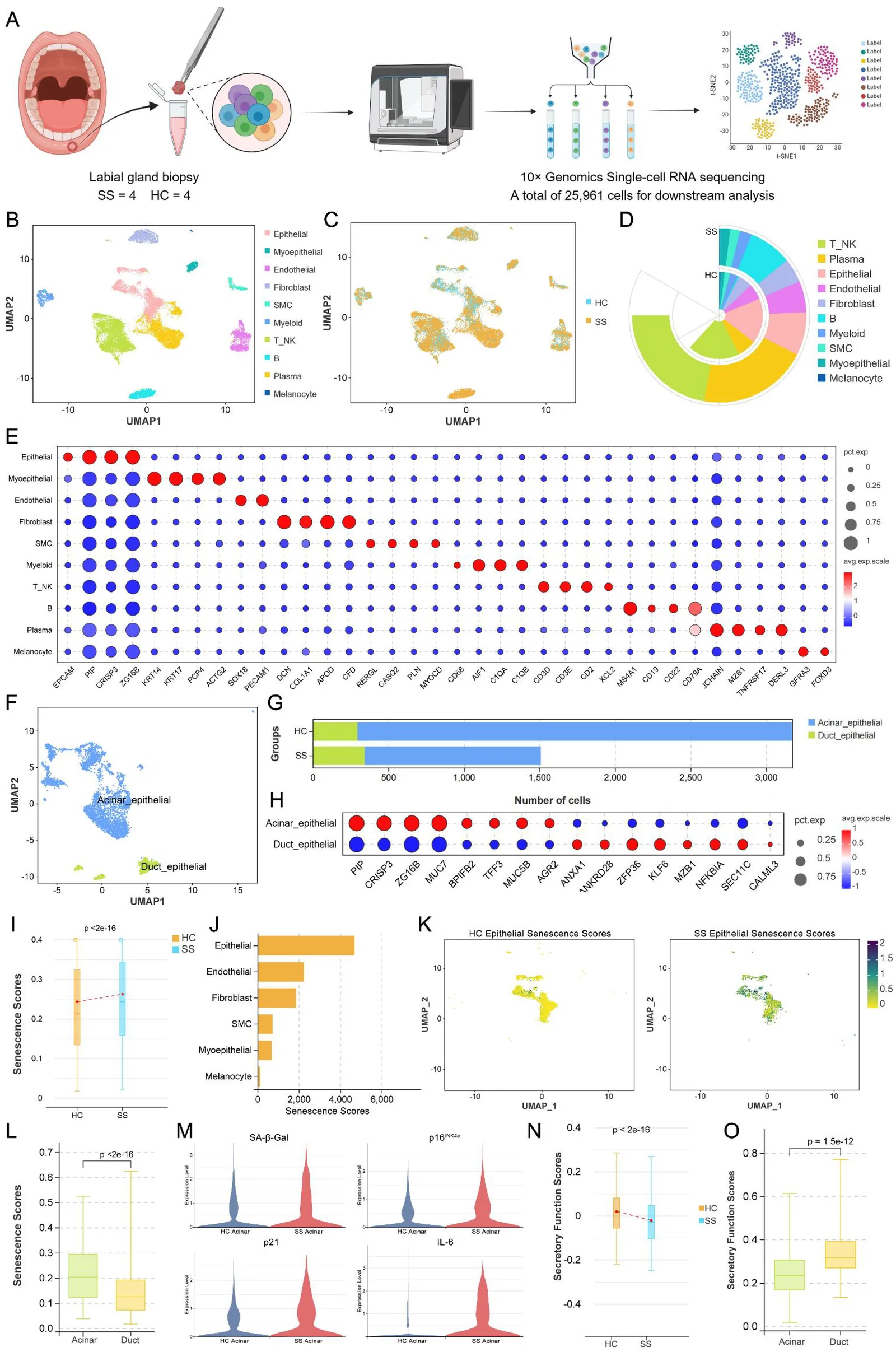
** ScRNA-seq revealed key features of acinar cell senescence and secretory dysfunction in SS labial glands. (A)** Schematic of the scRNA-seq workflow, showing the experimental process from labial gland biopsy collection to data analysis (created at https://BioRender.com). **(B-C)** UMAP plots showing the clustering of major cell types (B) and comparison between SS and HC groups (C), based on the expression of cluster-specific and marker genes. **(D)** Pie chart comparing the absolute numbers of different cell types in the labial glands across all samples. **(E)** Dot plot showing the expression of marker genes for each cell subpopulation. The size of the dot represents the percentage of cells expressing the gene (pct. exp), and the color intensity represents the average expression level (avg. exp scale) among expressing cells. **(F)** UMAP plot showing the subdivision of epithelial cells into acinar and ductal cells. **(G)** The comparison of the absolute numbers of acinar and ductal cells in SS and HC groups highlights a more significant reduction in acinar cells in SS patients. **(H)** The expression of marker genes for acinar and ductal cells in the labial glands of SS patients and HCs. The size of the dot represents the percentage of cells expressing the gene (pct. exp), and the color intensity represents the average expression level (avg. exp scale) among expressing cells. **(I)** Boxplot displaying the senescence scores calculated in SS and HC groups, with significantly higher scores in the SS group. **(J)** Bar graph comparing the senescence scores of resident tissue cells, showing the highest senescence scores in epithelial cells in SS patients. **(K)** UMAP plot comparing senescence scores of epithelial cells in SS and HC groups, indicating higher scores in SS patients. **(L)** Boxplot comparing senescence scores between acinar and ductal cells in SS group, showing significantly higher scores in acinar cells. **(M)** Violin plots showing gene expression levels of four senescence markers (SA-β-Gal, p16^INK4a^, p21, and IL-6) in acinar cells from SS and HC patients, indicating significant increases in SS. **(N)** Boxplot comparing the secretory function scores of epithelial cells in SS and HC groups, with significantly lower scores in SS patients. **(O)** Boxplot comparing secretory function scores between acinar and ductal cells in SS group, showing a more severe impairment in acinar cells. pct. exp., percentage of cells with expression. avg. exp scale, average expression scaled.

**Figure 2:**
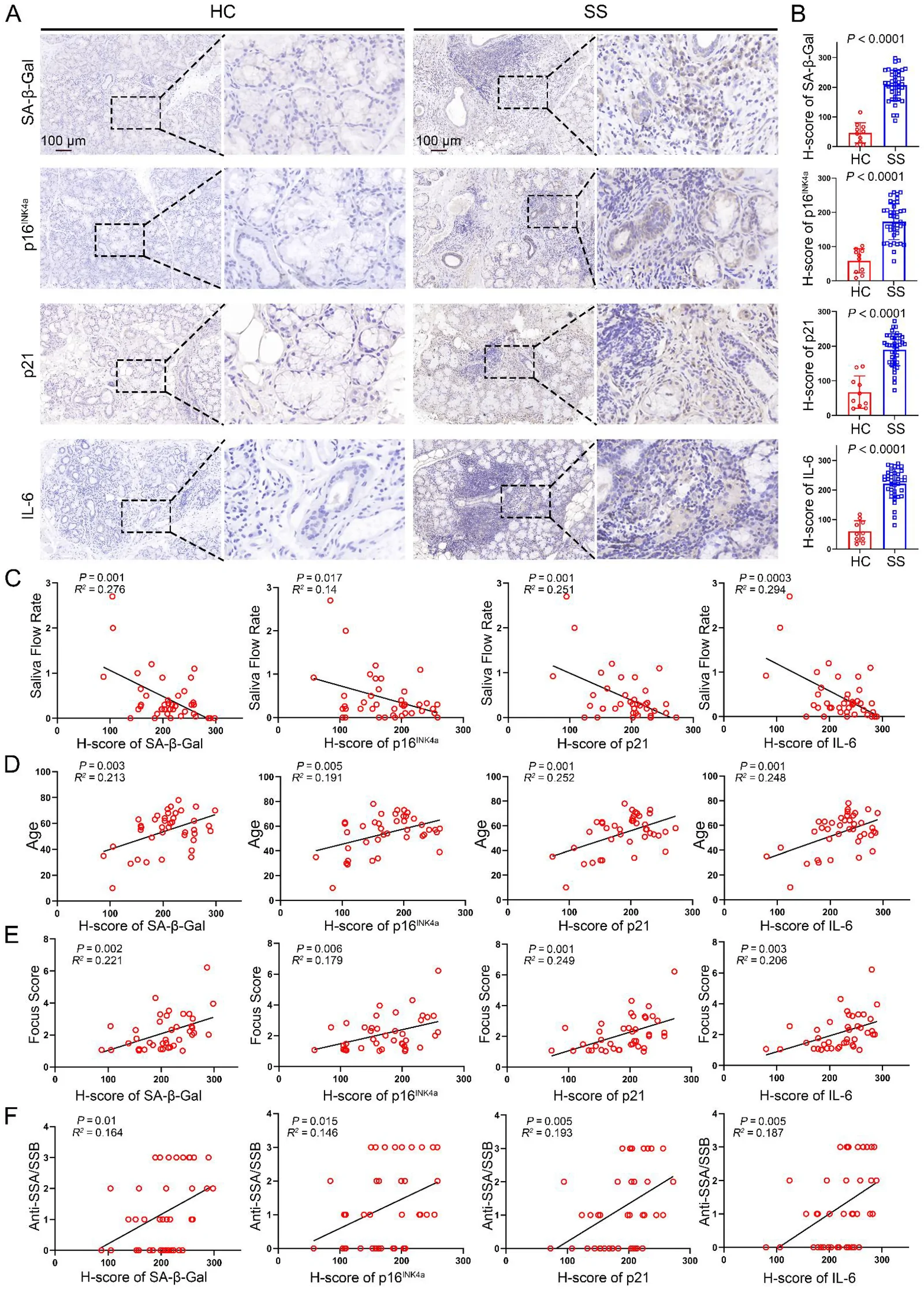
** Histological validation confirms labial gland senescence and its correlation with clinicopathological features in SS patients. (A)** Representative IHC images showing the high expression levels of senescence markers SA-β-Gal, p16^INK4a^, p21, and IL-6 in labial glands of SS patients (scale bar, 100 μm). **(B)** Quantitative analysis of histoscores reveals significantly higher expression levels of SA-β-Gal, p16^INK4a^, p21, and IL-6 in SS patients compared to HCs, confirming the presence of senescent phenotypes in SS labial glands. **(C-F)** Spearman's rank correlation analysis shows that the histoscores of senescence markers (SA-β-Gal, p16^INK4a^, p21, and IL-6) are negatively correlated with the unstimulated whole saliva flow rate in SS patients (C), and positively correlated with patient age (D), lymphocytic focus scores (E), and serum anti-SSA/SSB levels (F), suggesting a close relationship between labial gland senescence and SS progression.

**Figure 3:**
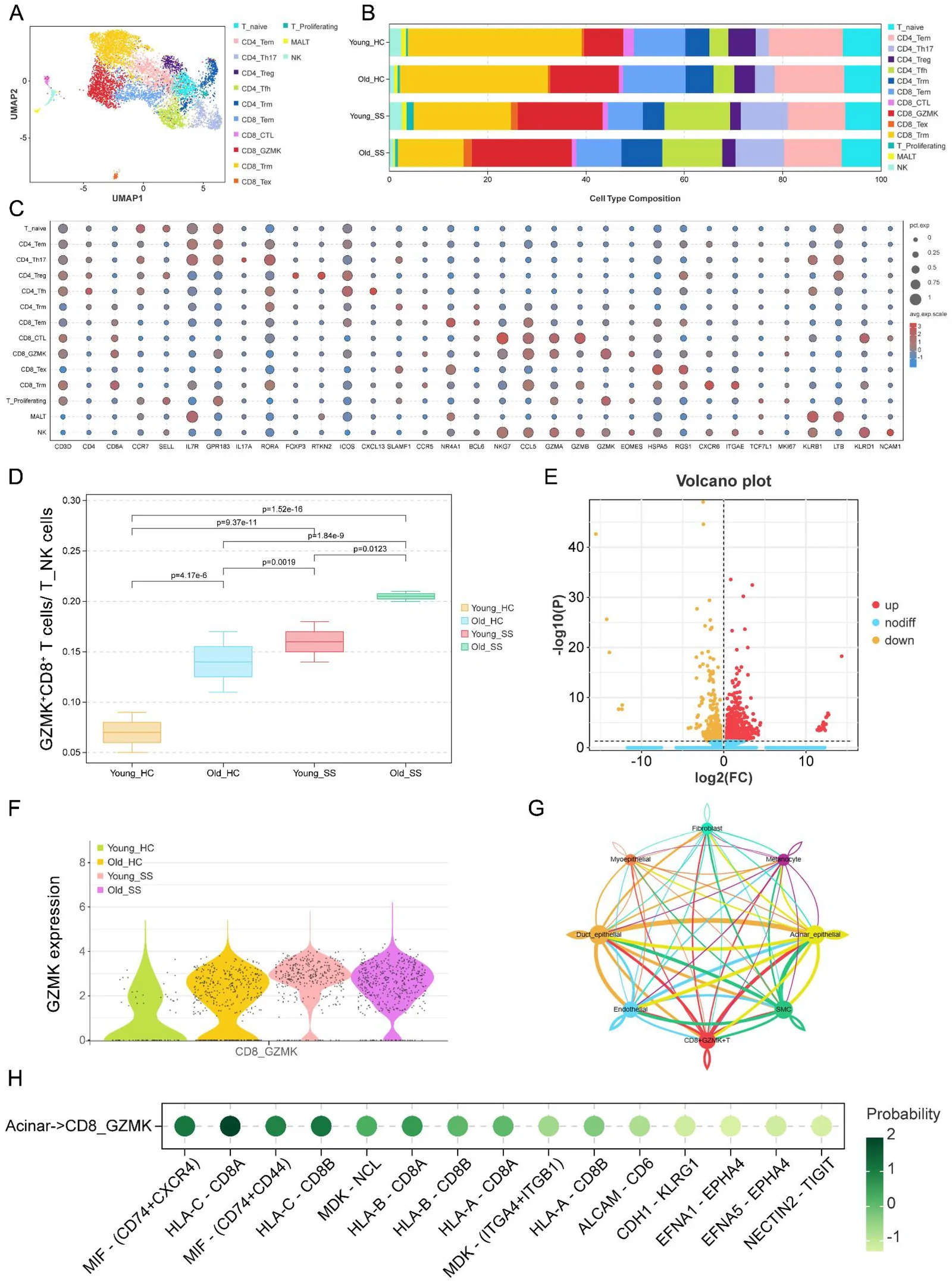
** Senescence-associated GZMK⁺CD8⁺ T cells exhibit significant interactions with SS acinar cells. (A)** UMAP plot showing the re-annotation and subclassification of T/NK cell populations into 14 distinct subsets from labial gland samples. **(B)** Bar graph displaying the proportional composition of T cell subsets in young and elderly SS patients and age- and sex-matched HCs, highlighting the increased proportion of GZMK⁺CD8⁺ T cells in SS patients. **(C)** The key markers associated with different T cell subsets in SS and HC groups. **(D)** Boxplot showing the proportion of GZMK⁺CD8⁺ T cells in the young and elderly SS groups compared to young and elderly HC groups, with the highest abundance observed in the elderly SS group. **(E)** Volcano plot of DEGs in GZMK⁺CD8⁺ T cells from SS patients compared to HCs, showing significantly upregulated functional genes in SS. **(F)** Boxplots showing the expression levels of GZMK in GZMK⁺CD8⁺ T cells from young and elderly SS patients and HCs, with significantly higher expression in SS groups, especially in the elderly SS group. **(G)** CellChat analysis of intercellular communication between GZMK⁺CD8⁺ T cells and various resident tissue cell types in labial glands, highlighting significant interactions with acinar cells. **(H)** Top 15 ligand-receptor pairs involved in the interaction between GZMK⁺CD8⁺ T cells and acinar cells, demonstrating the close communication between these cell types.

**Figure 4:**
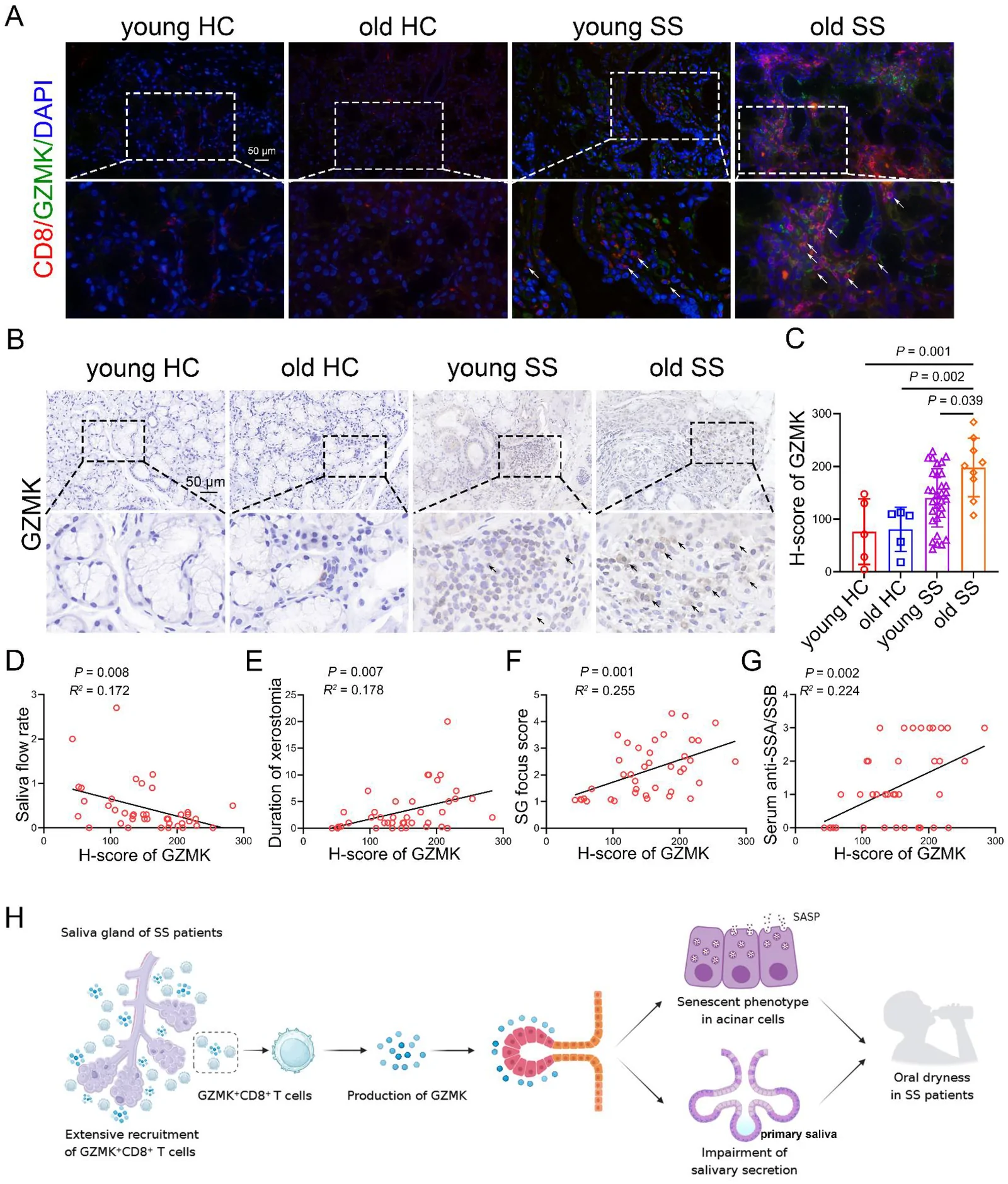
** GZMK⁺CD8⁺ T cells correlate with salivary dysfunction and disease severity in SS patients. (A)** IF staining shows infiltration of GZMK⁺CD8⁺ T cells around acinar cells in labial gland tissue from both elderly and young SS patients, with higher infiltration levels in the elderly SS group compared to HCs (scale bar, 50 μm). **(B-C)** Representative IHC images and histoscore quantification reveal significantly higher GZMK expression in labial glands of both young and elderly SS patients compared to HCs, with the elderly SS group showing the highest GZMK histoscore (scale bar, 50 μm). Data are presented as mean ± SD. Statistical significance was assessed using one-way ANOVA followed by Tukey's multiple-comparison test. Exact *P* values are indicated in the figure. **(D-G)** Spearman's rank correlation analysis shows that GZMK histoscores are negatively correlated with the unstimulated whole saliva flow rate in SS patients **(D)**, and positively correlated with the duration of xerostomia **(E)**, lymphocytic focus scores** (F)**, and serum anti-SSA/SSB levels **(G)**, indicating a close association between GZMK levels and SS severity. **(H)** The schematic illustrates that during the progression of SS, GZMK⁺CD8⁺ T cells produce GZMK and act on acinar cells, inducing senescence-associated phenotypes and impairing their salivary secretion function, ultimately contributing to the development of xerostomia in SS patients (created at https://BioRender.com).

**Figure 5:**
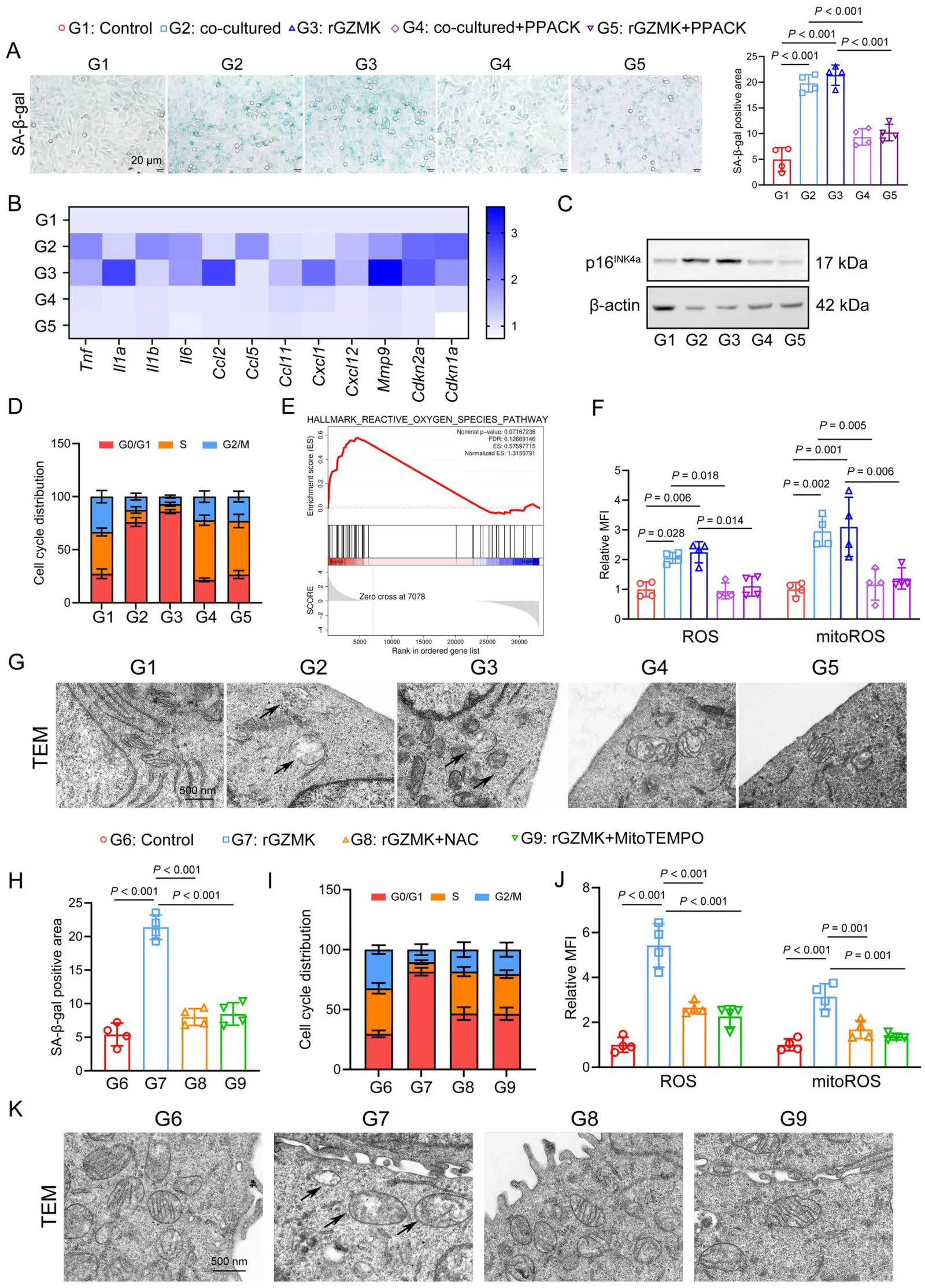
** GZMK⁺CD8⁺ T cells induce mitochondrial dysfunction in acinar cells, leading to senescent phenotypes.** For **(A-G)**, SMG-C6 cells were assigned to the following groups: G1, control; G2, co-culture with GZMK⁺CD8⁺ T cells; G3, rGZMK; G4, GZMK⁺CD8⁺ T cell co-culture plus PPACK; and G5, rGZMK plus PPACK. **(A)** Representative images of senescence-associated β-galactosidase (SA-β-gal) staining and quantification of the SA-β-gal positive area. Scale bar, 20 μm. **(B)** Heatmap showing the relative expression of senescence- and senescence-associated secretory phenotype-related genes, including *Tnf, Il1a, Il1b, Il6, Ccl2, Ccl5, Cxcl11, Cxcl1, Cxcl12, Mmp9, Cdkn2a,* and* Cdkn1a*, in the indicated groups. **(C)** Representative western blot image of p16^INK4a^ protein expression. **(D)** Cell-cycle distribution determined by propidium iodide staining and flow cytometry. The proportions of cells in the G0/G1, S, and G2/M phases are shown. **(E)** GSEA showing positive enrichment of the HALLMARK_REACTIVE_ OXYGEN_SPECIES_PATHWAY in SS acinar cells compared with HC acinar cells. **(F)** Quantification of intracellular reactive oxygen species (ROS) and mitochondrial ROS (mitoROS) levels by flow cytometry, expressed as relative mean fluorescence intensity (MFI). **(G)** Representative transmission electron microscopy (TEM) images showing mitochondrial ultrastructure in the indicated groups. Arrows indicate swollen or structurally disrupted mitochondria. Scale bar, 500 nm. For **(H-K)**, SMG-C6 cells were assigned to the following groups: G6, control; G7, rGZMK; G8, rGZMK and NAC; and G9, rGZMK and MitoTEMPO. **(H)** Quantification of the SA-β-gal-positive area after antioxidant treatment. Cell-cycle distribution **(I)** and quantitative analysis of intracellular ROS and mitoROS levels **(J)** in the indicated groups. **(K)** Representative TEM images showing mitochondrial ultrastructure following NAC or MitoTEMPO treatment. Arrows indicate damaged mitochondria. Scale bar, 500 nm. Data are presented as mean ± SD. Statistical comparisons were performed using one-way ANOVA followed by Tukey's multiple-comparison test. Exact *P* values are indicated in the figure.

**Figure 6:**
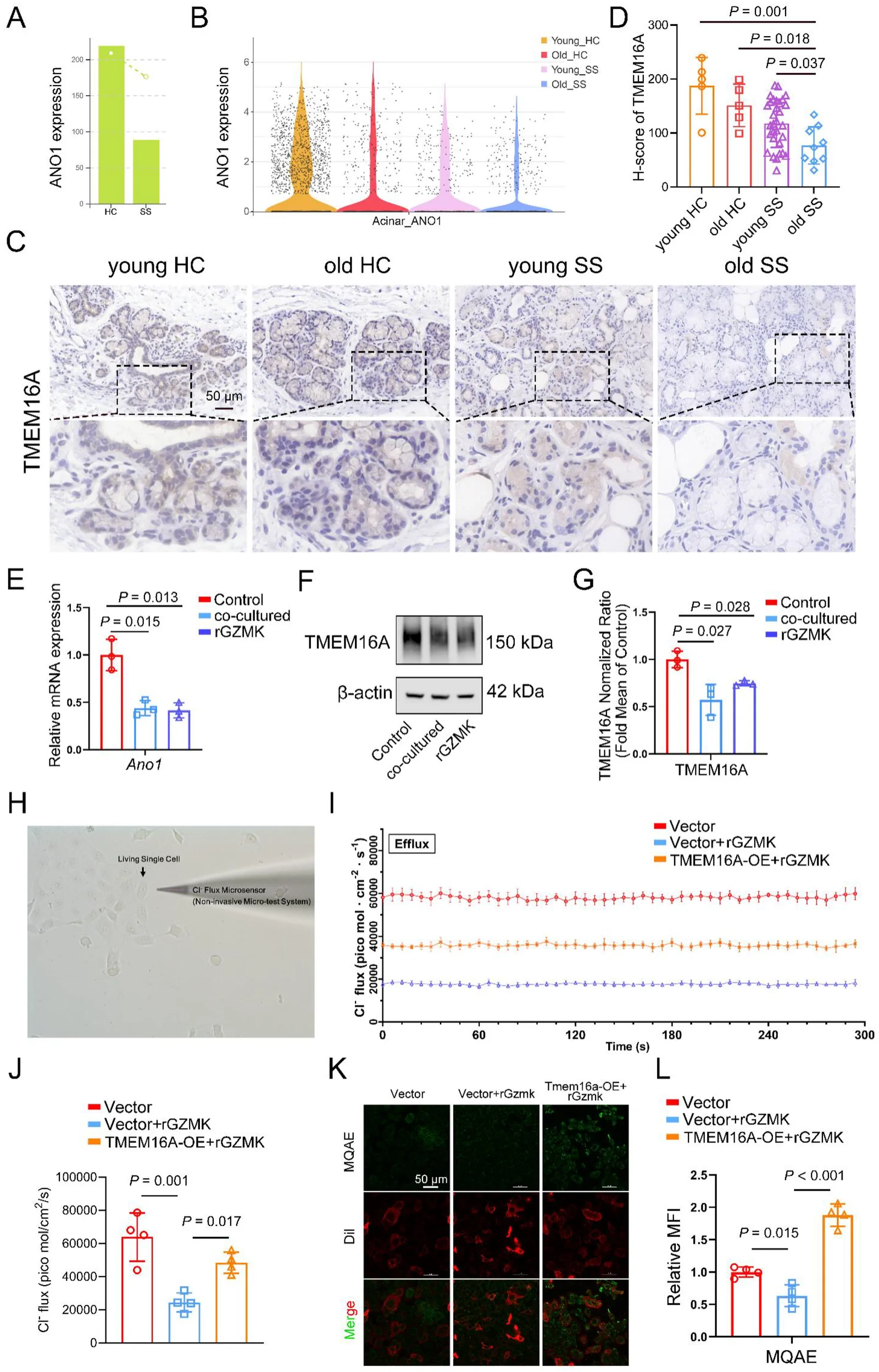
** GZMK⁺CD8⁺ T cells impair Cl^⁻^ transport and salivary secretion by inhibiting TMEM16A in acinar cells. (A-B)** scRNA-seq analysis reveals significant downregulation of TMEM16A (*ANO1*) expression in SS acinar cells, with the lowest expression in elderly SS patients compared to HCs. **(C-D)** Representative IHC images and quantitative analysis show significantly reduced TMEM16A expression in labial gland tissue of both young and elderly SS patients, with the lowest histoscores observed in the elderly SS group (scale bar, 50 μm). **(E)** qPCR analysis shows that TMEM16A mRNA expression (*Ano1*) is significantly reduced in both GZMK⁺CD8⁺ T cell co-cultured groups and rGZMK-treated groups. **(F-G)** Western blot assay and quantitative analysis demonstrate that TMEM16A protein levels in SMG-C6 cells are significantly decreased after co-culturing with GZMK⁺CD8⁺ T cells or treatment with rGZMK. **(H)** Representative NMT images showing the detection of Cl^⁻^ efflux in SMG-C6 cells. **(I)** NMT-based real-time monitoring of Cl^⁻^ efflux in SMG-C6 cells, with a 6-second interval over a 294-second duration. **(J)** Bar graph quantifying the average Cl^⁻^ efflux rate in SMG-C6 cells, showing a significant decrease in rGZMK-treated cells to approximately one-third of the rate observed in control cells. In contrast, overexpression of TMEM16A in SMG-C6 cells significantly restored the Cl^⁻^ efflux rate, reaching levels twice those in the rGZMK treatment group. **(K-L)** CLSM analysis of intracellular Cl^⁻^ concentrations showed a significant decrease in MQAE fluorescence intensity in rGZMK-treated cells, indicating impaired Cl^⁻^ efflux. In the TMEM16A overexpression group, MQAE fluorescence intensity recovered, suggesting restored Cl^⁻^ transport (scale bar, 50 μm).

**Figure 7:**
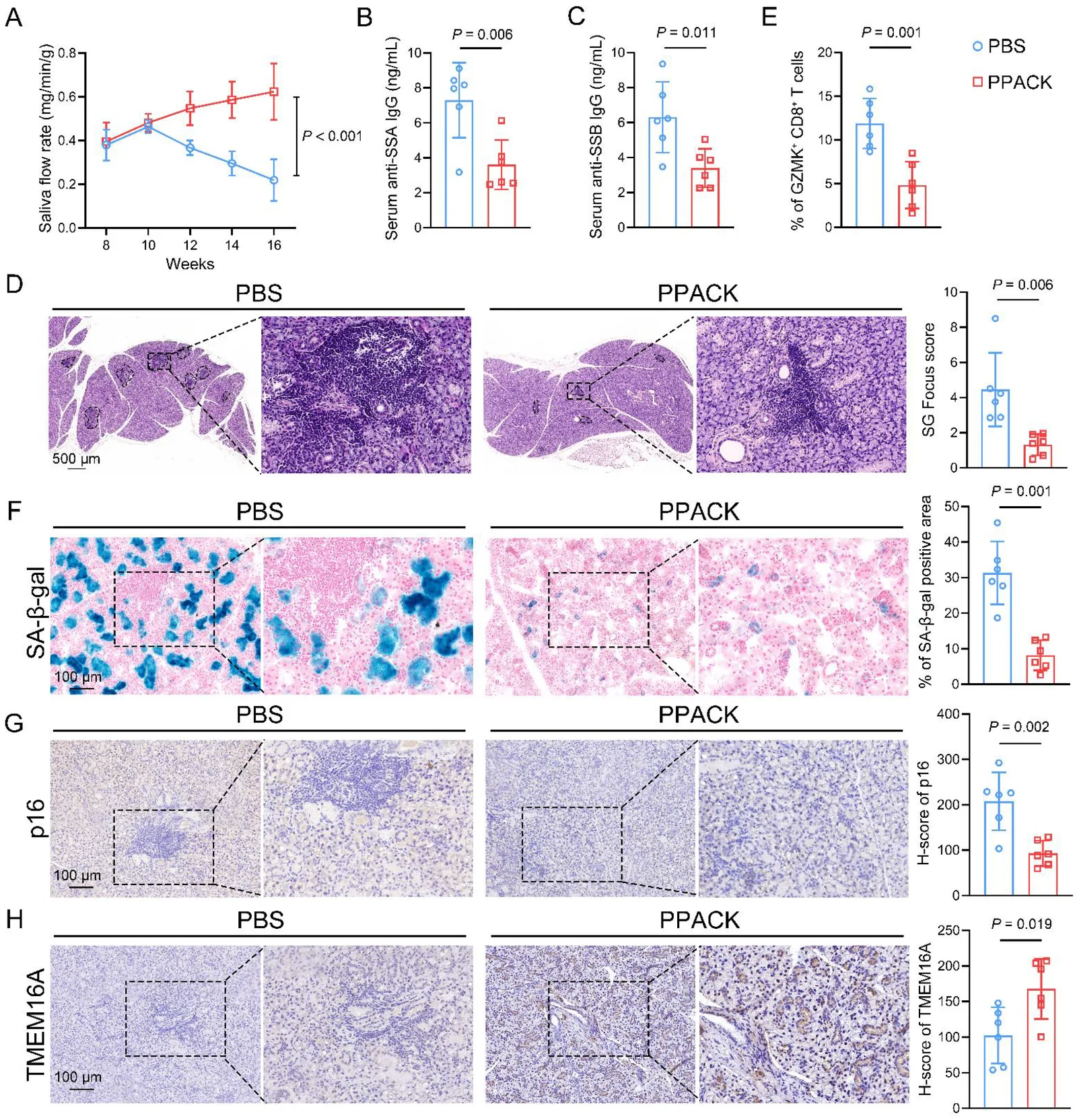
** Pharmacological inhibition of GZMK ameliorates glandular dysfunction and attenuates senescence-associated changes in NOD mice. (A)** Longitudinal measurement of stimulated salivary flow rates. Salivary secretion progressively declined in PBS-treated mice but was preserved and increased following PPACK treatment. Serum anti-SSA IgG **(B)** and anti-SSB IgG **(C)** concentrations were measured by ELISA at the experimental endpoint. **(D)** Representative H&E images of submandibular glands and quantitative analysis of salivary-gland focus scores. Dashed boxes indicate the regions shown at higher magnification. Scale bar, 500 μm. **(E)** Flow-cytometric quantification of GZMK⁺CD8⁺ T cells in submandibular glands. **(F)** Representative SA-β-gal staining of submandibular gland and quantification of the SA-β-gal-positive area relative to the analyzed glandular parenchymal area. Blue staining indicates SA-β-gal activity. Scale bar, 100 μm. Representative immunohistochemical staining and H-score quantification of p16^INK4a^ **(G)** and TMEM16A **(H)** in submandibular gland tissues. Dashed boxes indicate the regions shown at higher magnification. Scale bars, 100 μm. *n*=6 mice per group. Data are presented as mean ± SD. Statistical comparisons were performed using one-way ANOVA followed by Tukey's multiple-comparison test. Exact *P* values are shown in the figure.

## Data Availability

All the data generated during the current study are available from the corresponding author on reasonable request. The scRNA-seq dataset has been deposited in BioProject under accession number PRJCA068936 in China National Genomics Data Center.

## Abbreviations

SS: Sjögren's syndrome
AECG: American European Consensus Group
HCs: healthy controls
GZMK: granzyme K
scRNA-seq: single-cell RNA sequencing
DEGs: differentially expressed genes
GSEA: Gene Set Enrichment Analysis
SASP: senescence-associated secretory phenotype
SA-β-Gal: senescence-associated β-galactosidase
Cl^⁻^: chloride
ROS: reactive oxygen species
mitoROS: mitochondrial ROS
TEM: transmission electron microscopy
CLSM: confocal laser scanning microscopy
NMT: non-invasive micro-test technology
