# Supplementary material for: GZMK⁺CD8⁺ T Cells Induce Acinar Cell Senescence and Salivary Secretion Impairment in Sjögren's Syndrome via Mitochondrial Dysfunction and TMEM16A Chloride Channel Disruption
Source: Int J Biol Sci. 2026 Oct 2;22(15):8591–613. doi: 10.7150/ijbs.134478 (PMC13685753; doi:10.7150/ijbs.134478)
Supplement: Supplementary file 1 [file ijbsv22p8591s1.pdf]

Supplementary Figure

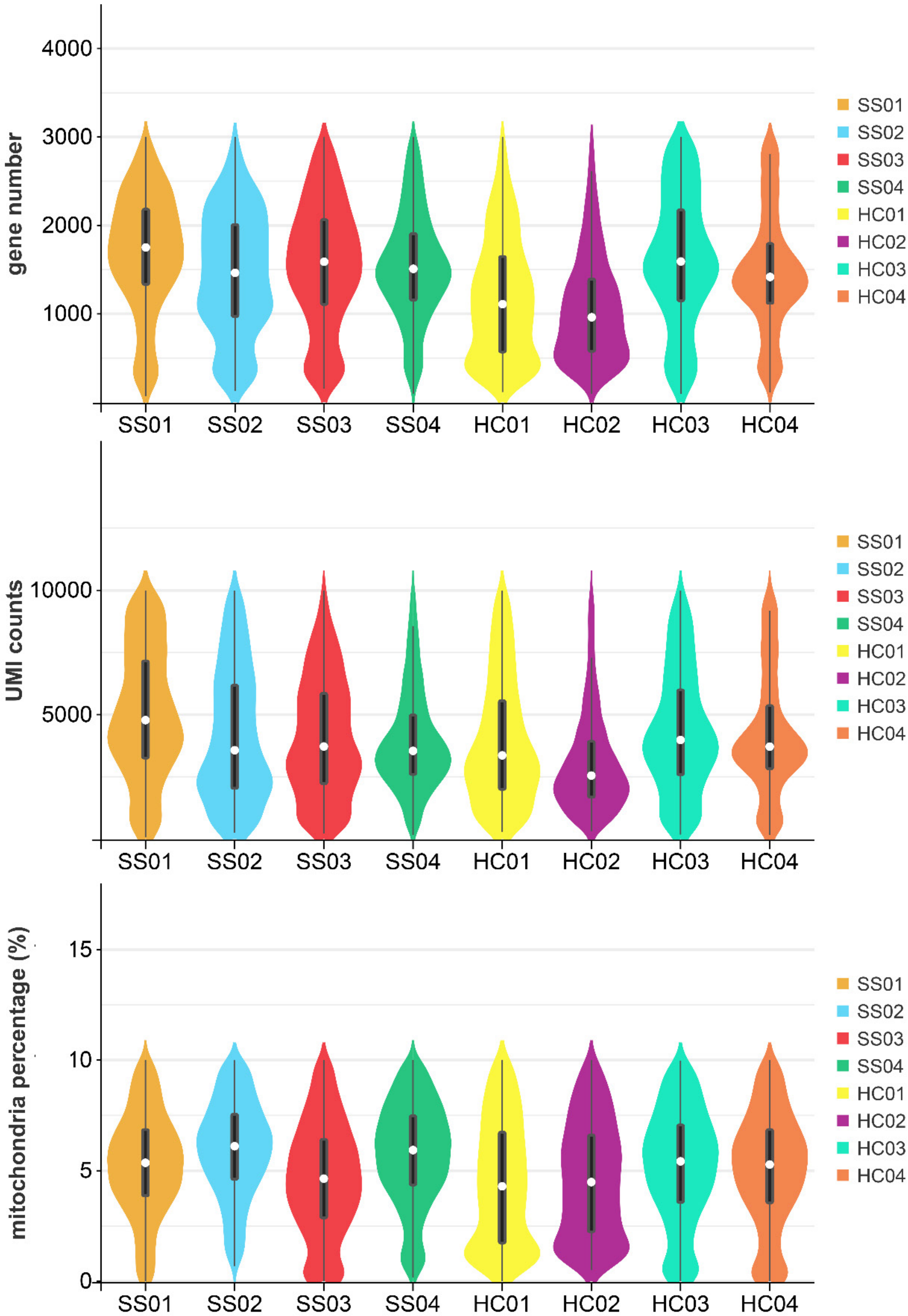

Figure S1. Quality control overview of scRNA-seq samples, including effective genes, transcripts, and mitochondrial percentage.

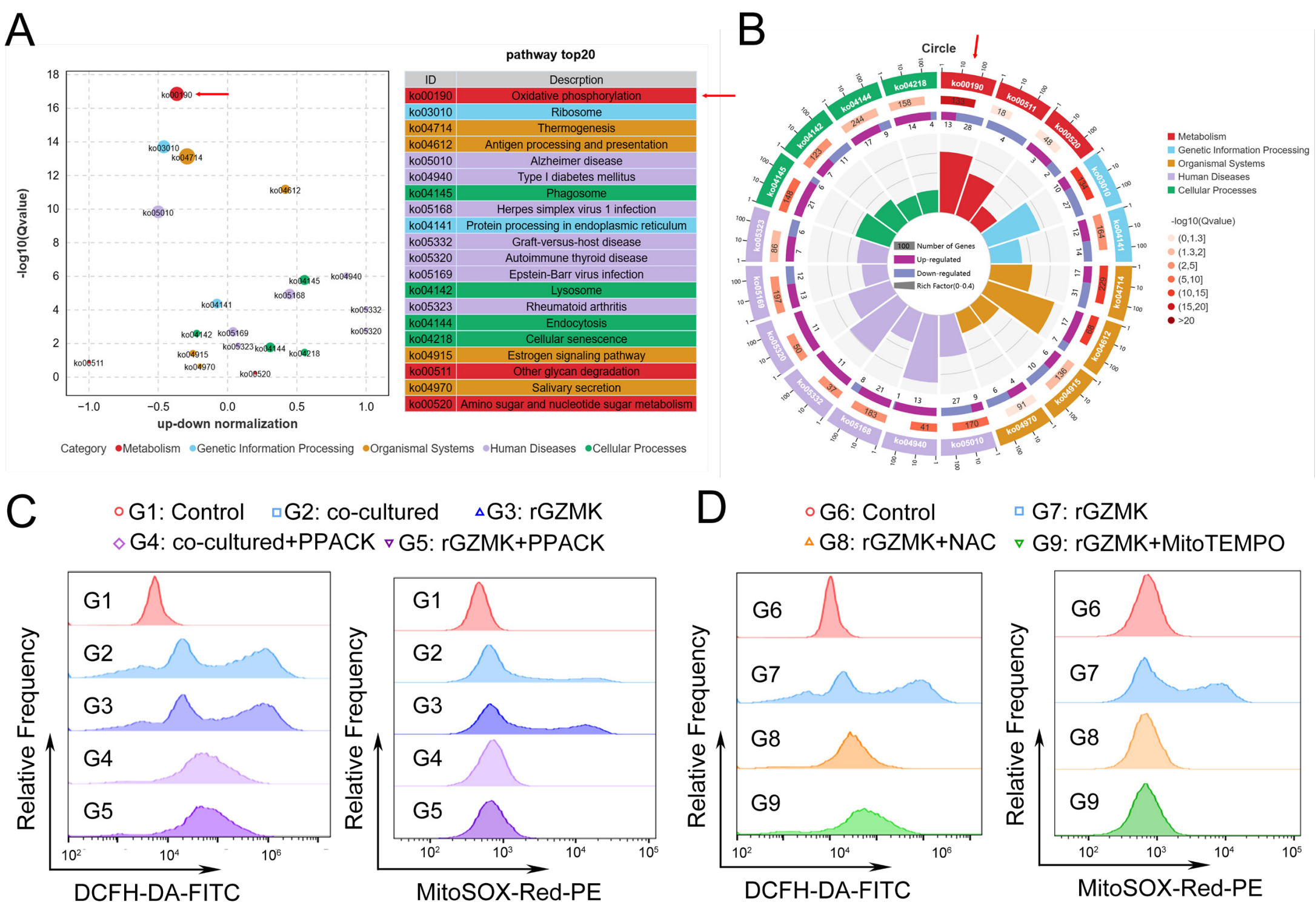

Figure S2. A, B. KEGG pathway analysis highlights the enrichment of the oxidative phosphorylation pathway, showing significant downregulation of 28 DEGs and upregulation of only 13 DEGs, suggesting mitochondrial dysfunction in SS acinar cells. **C.** Representative DCFH-DA (left panel) and MitoSOX (right panel) flow cytometry images in SMG-C6 cells treated with 20 ng/mL rGZMK and 10 µg/mL PPACK for 24 h. **D.** Representative DCFH-DA (left panel) and MitoSOX (right panel) flow cytometry images in SMG-C6 cells treated with 20 ng/mL rGZMK, 5 mM NAC and 10 µM MitoTEMPO for 24 h.

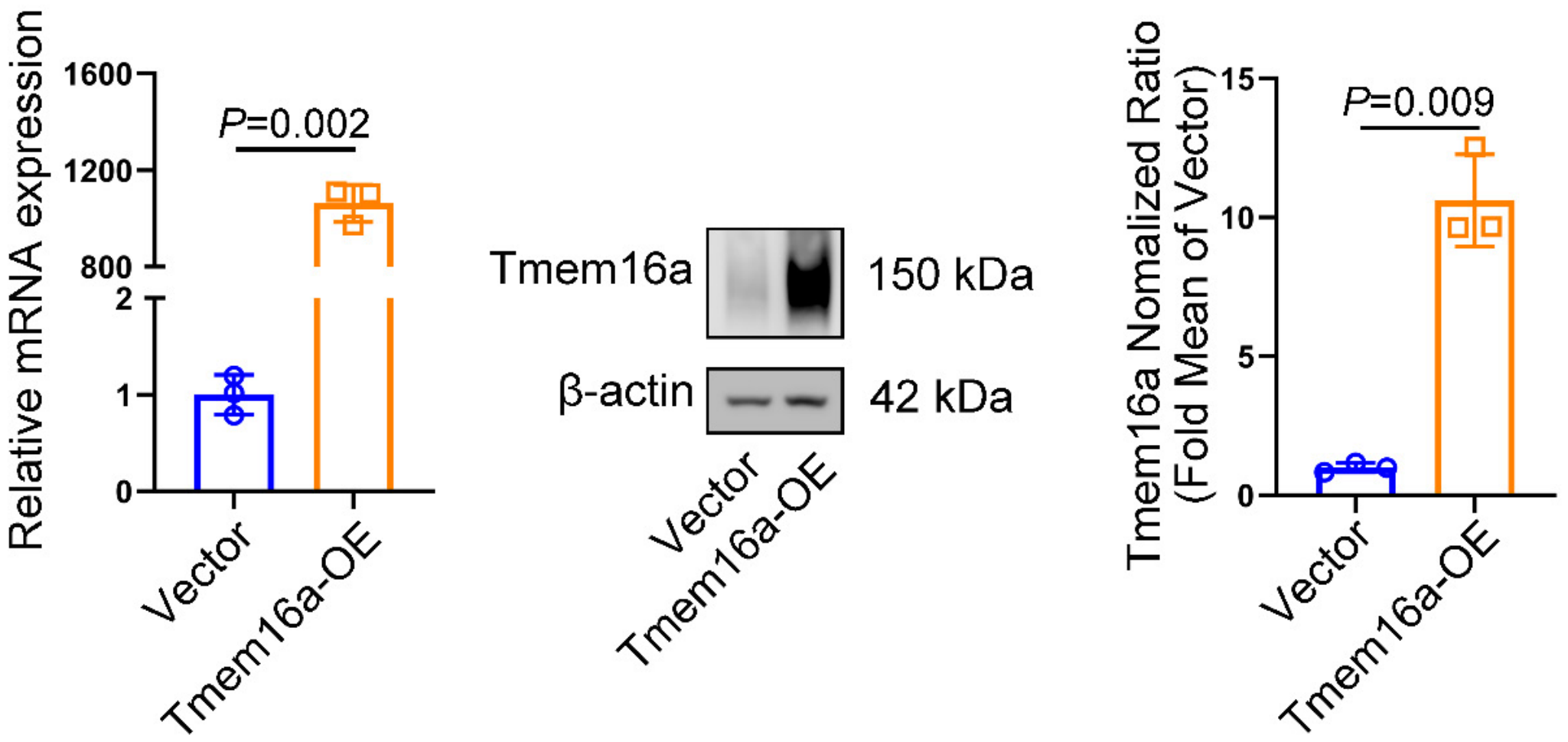

Figure S3. qRT-PCR and Western blot assays confirm the successful overexpression of Tmem16a at both the mRNA and protein levels.

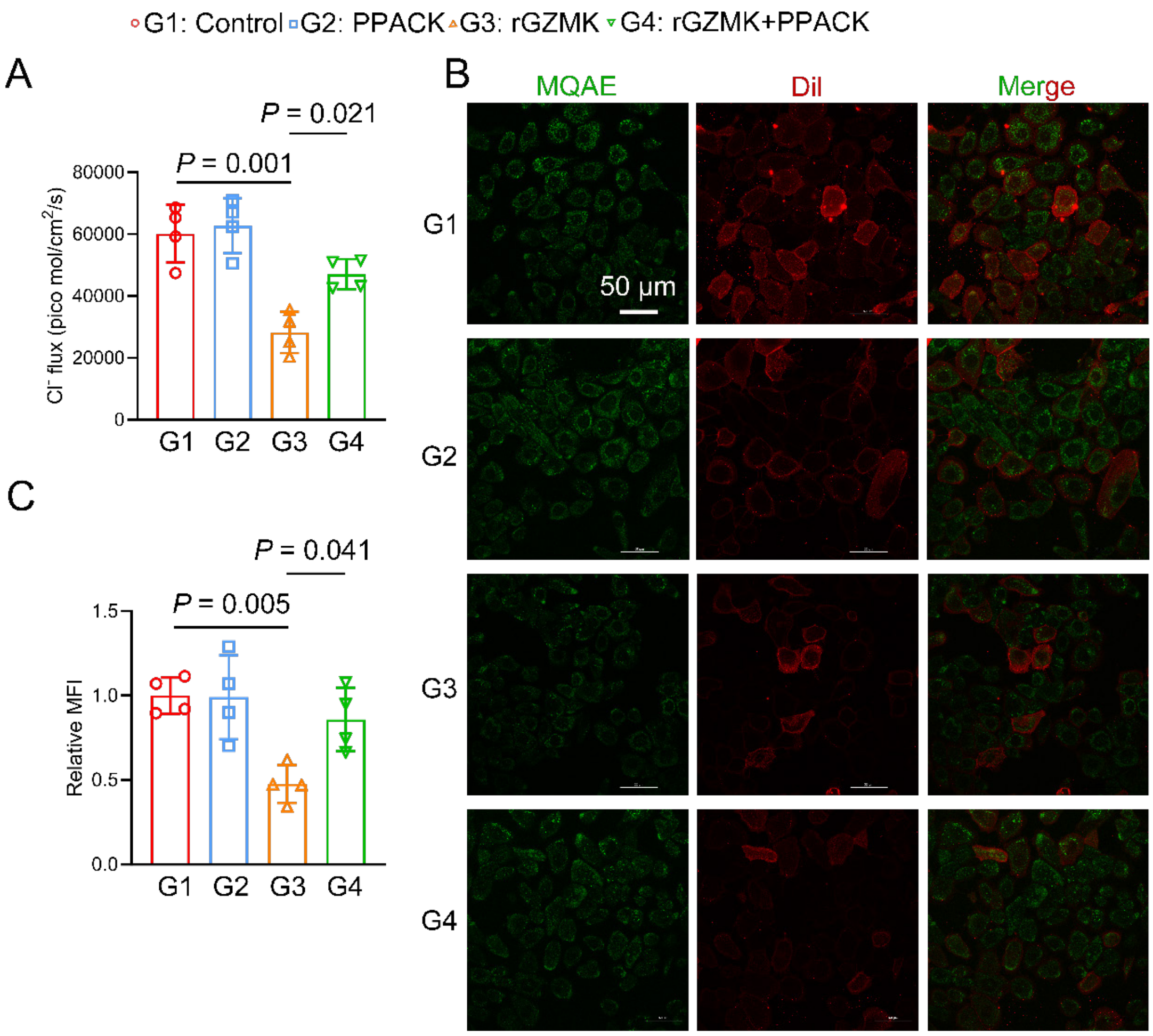

Figure S4. PPACK attenuates rGZMK-induced chloride transport impairment in SMG-C6 cells.

SMG-C6 cells were assigned to four groups: control (G1), PPACK alone (G2), recombinant GZMK (rGZMK; G3), and rGZMK plus PPACK (G4). **(A)** Quantification of the Cl^- efflux rate measured using non-invasive micro-test technology. rGZMK markedly reduced Cl^- efflux compared with the control group, whereas PPACK treatment partially restored Cl^- efflux in rGZMK-treated cells. **(B)** Representative fluorescence images of intracellular chloride detected using the chloride-sensitive fluorescent probe MQAE. Cell membranes were labeled with DiI. Reduced MQAE fluorescence indicates increased intracellular Cl^- retention. Scale bar, 50 μm. **(C)** Quantification of the relative mean fluorescence intensity (MFI) of MQAE. rGZMK reduced MQAE fluorescence, whereas PPACK partially reversed this reduction. Each data point represents one independent experiment. Data are presented as mean ± SD from four independent experiments. Statistical significance was assessed using one-way ANOVA followed by Tukey's multiple-comparison test. Exact *P* values are indicated in the figure.

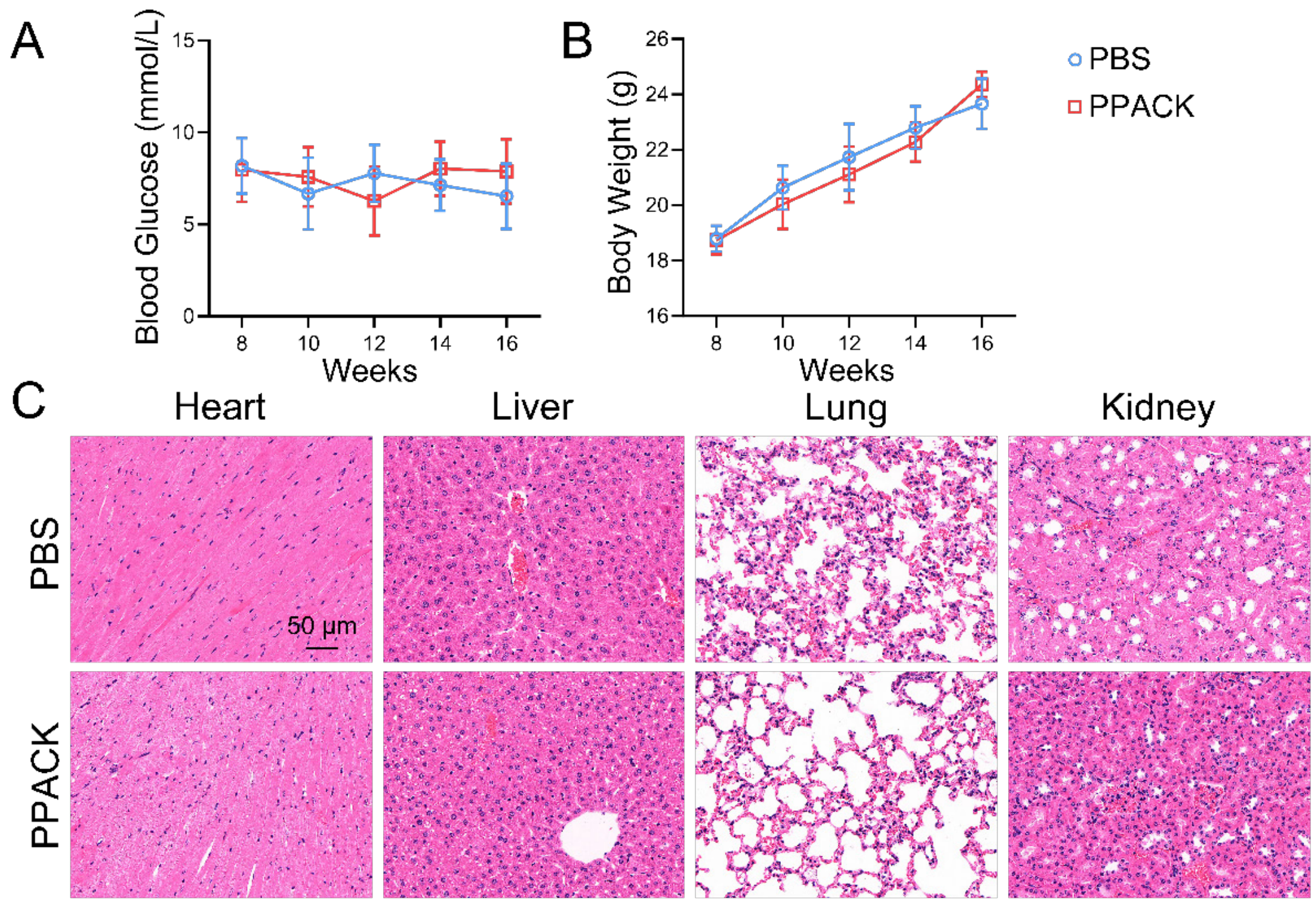

Figure S5. NOD mice received PBS or PPACK according to the indicated treatment protocol. **(A)** Blood glucose concentrations were monitored from 8 to 16 weeks of age and remained comparable between the PBS- and PPACK-treated groups. **(B)** Body weight was recorded over the same period. Both groups exhibited a similar age-dependent increase in body weight, with no evident treatment-related growth impairment. **(C)** Representative hematoxylin and eosin staining of the heart, liver, lungs, and kidneys collected at the experimental endpoint. No obvious inflammatory infiltration, tissue necrosis, or major structural abnormalities were observed in the PPACK-treated group compared with the PBS control group. Scale bar, 50 μm. Data are presented as mean ± SD, n=6 mice per group.

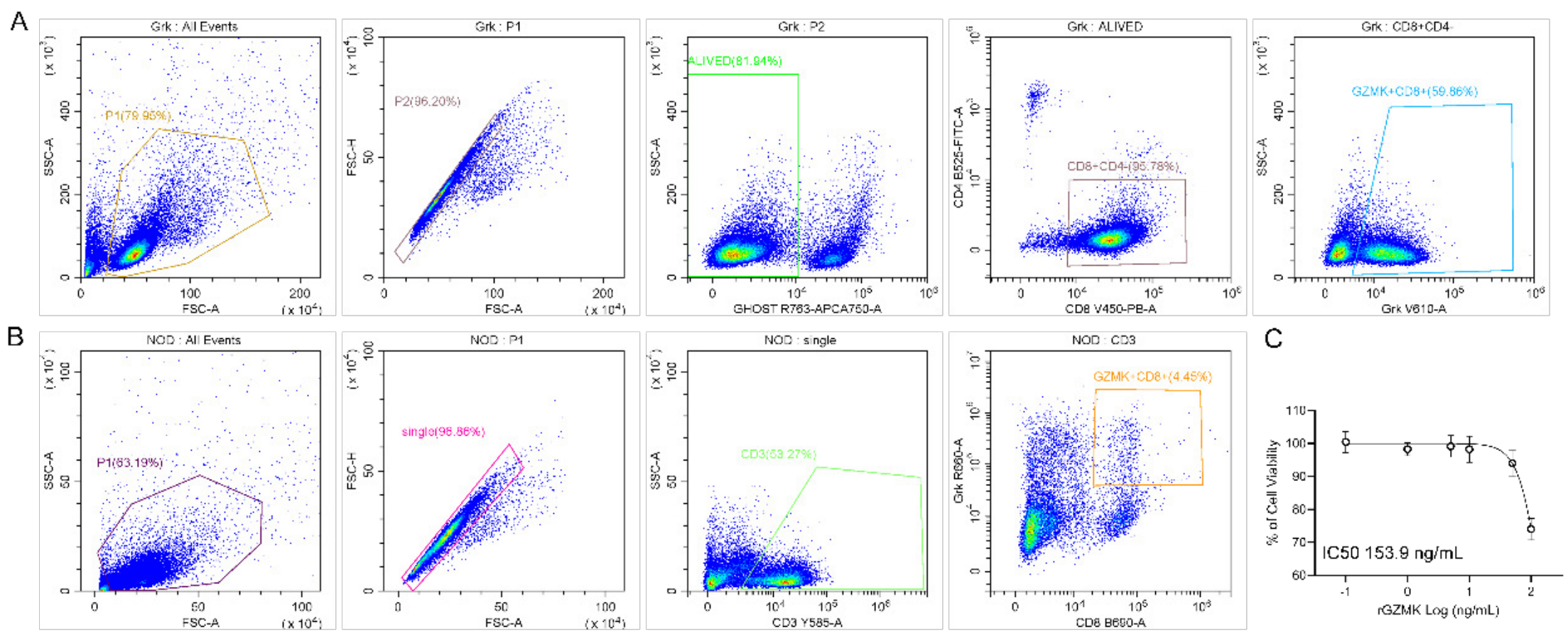

Figure S6. Flow cytometry gating strategies for GZMK$^+$CD8$^+$ T cells and determination of the rGZMK concentration range in SMG-C6 cells.

(A) Representative flow-cytometric gating strategy used to identify GZMK$^+$CD8$^+$ T cells for the co-culture experiments. Cells were sequentially gated based on forward- and side-scatter characteristics, singlets, viable cells, and the CD8$^+$CD4$^-$ population, followed by identification of GZMK$^+$ cells within the CD8$^+$ T-cell compartment.

(B) Representative gating strategy for detecting GZMK$^+$CD8$^+$ T cells in single-cell suspensions prepared from the submandibular glands of NOD mice. After exclusion of debris and doublets, CD3$^+$ T cells were selected, and GZMK$^+$CD8$^+$ T cells were subsequently identified within the CD3$^+$ population.

(C) Dose–response analysis of SMG-C6 cell viability following treatment with the indicated concentrations of recombinant GZMK. Cell viability was expressed relative to the untreated control, and nonlinear regression yielded an estimated half-maximal inhibitory concentration of 153.9 ng/mL. Data are presented as mean ± SD from 3 independent experiments.

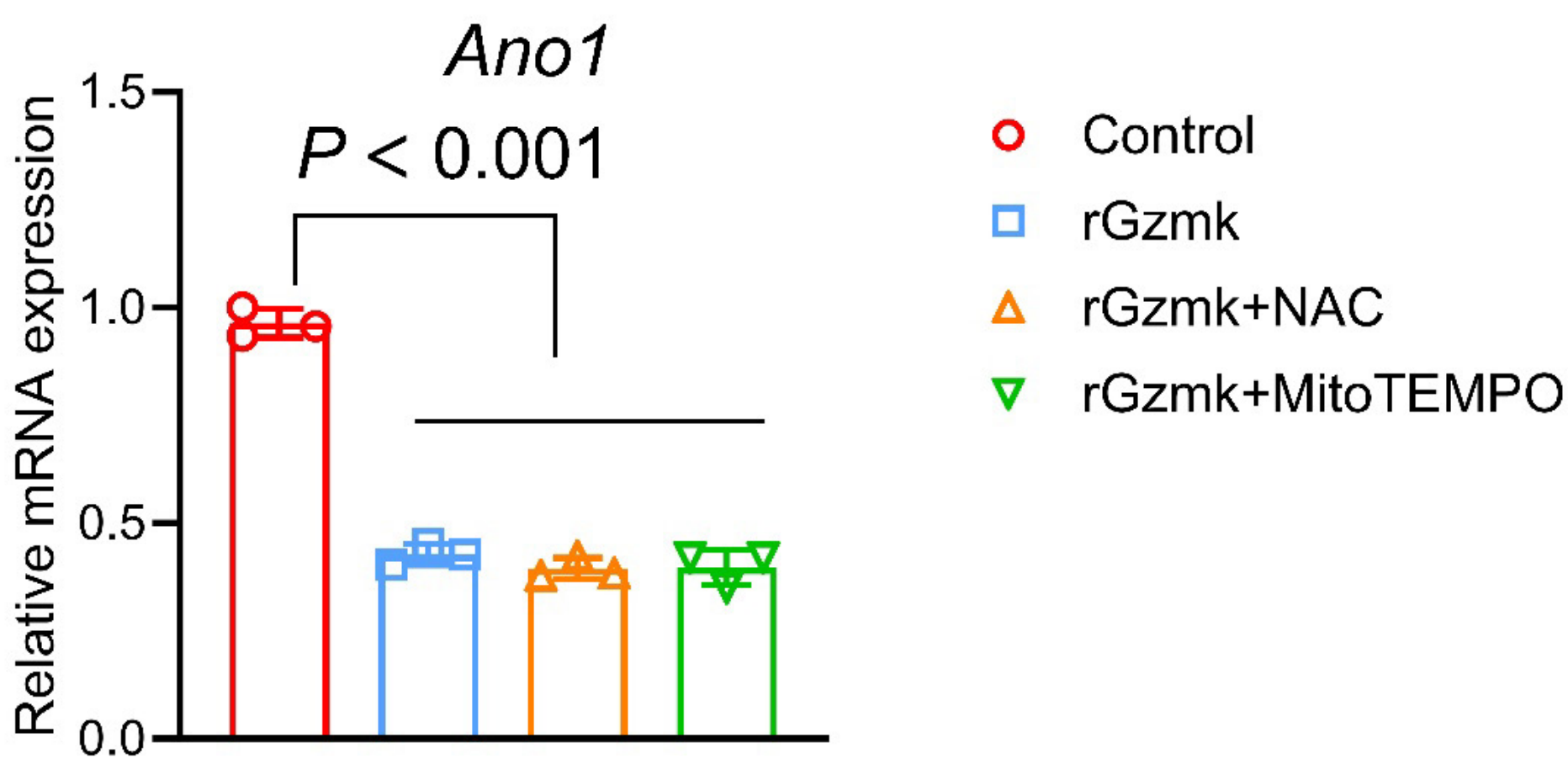

Figure S7. qPCR analysis shows that neither NAC nor MitoTEMPO treatment restored the reduced TMEM16A mRNA expression (*Ano1*) induced by recombinant GZMK.

Supplementary Table

Table S1. Clinical characteristics of SS and HC subjects for labial gland tissues single-cell RNA sequencing.

	Age (years)	Sex	Focus score of ≥1	UWSFR (mL/10 min)	Anti-SSA/SSB positive	Head and neck radiation history
SS1	23	F	Y	0.5	Y	N
SS2	31	F	Y	0.3	Y	N
SS3	65	F	Y	<0.1	Y	N
SS4	67	F	Y	0.1	Y	N
HC1	24	F	NA	NA	NA	N
HC2	28	F	NA	NA	NA	N
HC3	65	F	NA	NA	NA	N
HC4	68	F	NA	NA	NA	N

SS, Sjögren's Syndrome. HC, healthy control. F, Female. Y, Yes. N, No. NA, not applicable. Focus score ⩾1, Labial salivary gland with focal lymphocytic focus scores ⩾1 foci per 4 mm^2. UWSFR, whole unstimulated saliva flow rate.

Inclusion and Exclusion Criteria for Sjögren's Syndrome Patients:

- **Inclusion Criteria:**

(1) Patients who meet the 2002 AECG classification criteria for Sjögren's Syndrome. (2) Patients who have not received any treatment, including systemic medications (e.g., steroids), and are not taking any medications that could affect the immune system due to other underlying diseases (3) Competent patients who voluntarily agree to participate in the study and provide written informed consent.

- **Exclusion Criteria:**

(1) Patients with other systemic diseases unrelated to Sjögren's Syndrome (e.g., sarcoidosis, amyloidosis, graft-versus-host disease, IgG4-related disease, etc.). (2) Patients with infectious or acute infectious diseases (e.g., active hepatitis C, HIV, etc.). (3) Patients with a history of head and neck radiation therapy. (4) Patients with contraindications for surgery. (5) Patients who are unwilling to participate.

Inclusion and Exclusion Criteria for Healthy Control Group:

- **Inclusion Criteria:**

(1) Clinically diagnosed with a mucous cyst of the labial gland, who are scheduled for surgical excision and are matched to Sjögren's Syndrome patients in terms of age and gender. (2) Patients with no history of systemic diseases and who are otherwise healthy. (3) No use of medications that may affect the immune system in the past year. (4) No history of infectious or acute infectious diseases. (5) Competent patients who voluntarily agree to participate in the study and provide written informed consent.

- **Exclusion Criteria:**

(1) Patients with infectious or acute infectious diseases. (2) Patients with contraindications for surgery. (3) Patients who are unwilling to participate.

Table S2. Clinical characteristics of SS and HC subjects for immunohistochemistry and immunofluorescence.

	Age (years)	Sex	Focus score	UWSFR (mL/10 min)	Anti-SSA/SSB	Head and neck radiation history
SS1	10	F	2.55	2.7	++	N
SS2	49	F	1.51	1.2	-	N
SS3	57	F	3.29	0.2	+++	N
SS4	61	F	1.23	0.3	+	N
SS5	68	F	1.19	0.4	++	N
SS6	32	F	2.83	0.2	+	N
SS7	78	F	2.5	0.5	+++	N
SS8	30	F	1.08	0.5	+	N
SS9	52	F	3.01	1.1	+	N
SS10	70	M	0.7	0.05	-	N
SS11	63	F	1.36	0	-	N
SS12	60	F	1.2	0.2	-	N
SS13	62	F	1.11	0.2	-	N
SS14	54	F	2.01	0	+++	N
SS15	73	F	3.52	0	+++	N
SS16	57	F	1.11	0.3	++	N
SS17	55	F	2.01	0.1	+	N
SS18	53	F	3.31	0.15	+++	N
SS19	55	F	1.09	0.26	-	N
SS20	66	F	4.0	0.1	+++	N
SS21	70	M	3.96	0	++	N

SS22	39	F	2.23	0.3	+++	N
SS23	63	F	1.77	0.2	-	N
SS24	32	F	2.31	1	+	N
SS25	29	F	1.48	0	+	N
SS26	51	F	1.01	0.6	-	N
SS27	64	F	2.31	0.2	-	N
SS28	42	F	1.06	2	-	N
SS29	73	F	2.12	0.04	+++	N
SS30	35	M	1.07	0.92	-	N
SS31	64	F	1.47	0.3	+	N
SS32	53	F	1.11	0.9	-	N
SS33	47	F	2.54	0.35	+	N
SS34	71	F	1.7	0.1	-	N
SS35	58	F	4.0	0	++	N
SS36	62	F	2.45	0.3	++	N
SS37	57	F	3.21	0.3	+	N
SS38	58	F	1.02	0.65	-	N
SS39	34	F	3.31	0.9	+++	N
SS40	68	F	1.33	0.27	-	N
HC1	6	F	NA	NA	NA	N
HC2	47	M	NA	NA	NA	N
HC3	34	F	NA	NA	NA	N
HC4	8	F	NA	NA	NA	N
HC5	62	F	NA	NA	NA	N
HC6	53	F	NA	NA	NA	N
HC7	50	M	NA	NA	NA	N
HC8	58	F	NA	NA	NA	N
HC9	55	F	NA	NA	NA	N
HC10	68	F	NA	NA	NA	N

SS, Sjögren's Syndrome. HC, healthy control. F, Female. Y, Yes. N, No. NA, not applicable. UWSFR, whole unstimulated saliva flow rate.

Inclusion and Exclusion Criteria for Sjögren's Syndrome Patients:

- **Inclusion Criteria:**

(1) Patients who meet the 2002 AECG classification criteria for Sjögren's Syndrome. (2) Patients who have not received any treatment, including systemic medications (e.g., steroids), and are not taking any medications that could affect the immune system due to other underlying diseases (3) Competent patients who voluntarily agree to participate in the study and provide written informed consent.

- **Exclusion Criteria:**

(1) Patients with other systemic diseases unrelated to Sjögren's Syndrome (e.g., sarcoidosis, amyloidosis, graft-versus-host disease, IgG4-related disease, etc.). (2) Patients with infectious or acute infectious diseases (e.g., active hepatitis C, HIV, etc.). (3) Patients with a history of head and neck radiation therapy. (4) Patients with contraindications for surgery. (5) Patients who are unwilling to participate.

Inclusion and Exclusion Criteria for Healthy Control Group:

- **Inclusion Criteria:**

(1) Clinically diagnosed with a mucous cyst of the labial gland, who are scheduled for surgical excision and are matched to Sjögren's Syndrome patients in terms of age and gender. (2) Patients with no history of systemic diseases and who are otherwise healthy. (3) No use of medications that may affect the immune system in the past year. (4) No history of infectious or acute infectious diseases. (5) Competent patients who

voluntarily agree to participate in the study and provide written informed consent.

- **Exclusion Criteria:**

(1) Patients with infectious or acute infectious diseases. (2) Patients with contraindications for surgery. (3) Patients who are unwilling to participate.

Table S3. List of antibodies used in this study

Antibody	Company	Cat#	Clone#	Reacts	Applications
GZMK	Invitrogen	PA5-50980	Poly	Human	IHC (1:800)<br>IF (1:400)
TMEM16A	Abcam	ab53212	Poly	Human<br>Mouse	IHC (1:400)<br>IHC (1:400)
CD8α	CST	70306	C8/144B	Human	IF (1:400)
$p16^{INK4a}$	Proteintech	10883	Poly	Human	IHC (1:800)
p21	Proteintech	10355	Poly	Human	IHC (1:400)
IL-6	Proteintech	21865	Poly	Human	IHC (1:400)
β-Gal	Proteintech	15518	Poly	Human	IHC (1:200)
TMEM16A	CST	10463	F9W4N	Rat	WB (1:1000)
$p16^{INK4a}$	Abclonal	A23882	Poly	Rat<br>Mouse	WB (1:2000)<br>IHC (1:400)
β-actin	Proteintech	20536-1-AP	Poly	Rat	WB (1:5000)
CD3	BioLegend	100205	17A2	Mouse	FC (1:400)
CD8α	BD	570256	53-6.7	Mouse	FC (1:400)
GZMK	Invitrogen	50-8897-42	G3H69	Mouse	FC (1:100)
CD8α	BD	561614	OX-8	Rat	FC (1:400)
CD4	BD	561834	OX-38	Rat	FC (1:400)
GZMK	BioLegend	370509	GM26E7	Rat	FC (1:100)

Table S4. Primer sequences for qRT-PCR analyses.

Gene name		Primer sequences (5′ to 3′)
Actb	Forward	5’- AGATGACCCAGATCATGTTTGAGA -3’
	Reverse	5’- GCATGAGGGAGCGCGTAA -3’
Cdkn2a	Forward	5’- CCCGAACACTTTCGGTCGTA -3’
	Reverse	5’- TGTCTAGGAAGCCCTCCCG -3’
Cdkn1a	Forward	5’- ATGTCCGACCTGTTCCACAC -3’
	Reverse	5’- CAGACGTAGTTGCCCTCCAG -3’
Il6	Forward	5’- CACTTCACAAGTCGGAGGCT -3’
	Reverse	5’- TCTGACAGTGCATCATCGCT -3’
Tnf	Forward	5’- CAGCAGATGGGCTGTACCTT -3’
	Reverse	5’- AAATGGCAAATCGGCTGACG -3’
Il1a	Forward	5’- TACTCATCGGGAGGAGACGA -3’
	Reverse	5’- TCTCCTTCAGCAACACAGGC -3’
Il1b	Forward	5’- CCTATGTCTTGCCCGTGGAG -3’
	Reverse	5’- CACACACTAGCAGGTCGTCA -3’
Ccl2	Forward	5’- TGATCCCAATGAGTCGGCTG -3’
	Reverse	5’- TGGACCCATTCCTTATTGGGG -3’
Ccl5	Forward	5’- GTGCCCACGTGAAGGAGTAT -3’
	Reverse	5’- GCGGTTCCTTCGAGTGACAA -3’
Ccl11	Forward	5’- AACAGATGCACGCTGAAAGC -3’
	Reverse	5’- GGCATCCTGGACCCACTTTT -3’
Cxcl1	Forward	5’- ACTCAAGAATGGTCGCGAGG -3’
	Reverse	5’- CTTGGGGACACCCTTTAGCA -3’
Cxcl12	Forward	5’- TGCCGATTCTTTGAGAGCCA -3’
	Reverse	5’- GGGCACAGTTTGGAGTGTTG -3’
Mmp9	Forward	5’- GATCCCCAGAGCGTTACTCG -3’
	Reverse	5’- GTTGTGGAAACTCACACGCC -3’
Ano1	Forward	5’- CACCTCTTTGACAATCCTGCC -3’
	Reverse	5’- TCACAGCTACTCACCTCCTCTT -3’

Table S5. Senescence-related Pathway Gene Sets.

Gene	Casella et al.	Freund et al.	SenSig	SenMayo	GenAge	CellAge	GSGene	De Cecco et al.	SeneQuest	Occurrence
IL6	F	T	F	T	T	T	T	T	T	7
IGFBP3	F	T	T	T	T	T	T	F	T	7
EGFR	F	T	F	T	T	T	T	T	T	7
SERPINE1	F	T	F	T	T	T	F	T	T	6
IGFBP1	F	T	F	T	F	T	T	T	T	6
IGFBP7	F	T	T	T	F	T	T	F	T	6
FAS	F	T	T	T	T	F	F	T	T	6
FGF2	F	T	T	T	F	F	T	T	T	6
VEGFA	F	T	F	T	T	T	F	T	T	6
CDKN1A	F	F	T	T	T	T	T	F	T	6
CDKN2A	F	F	T	T	T	T	T	F	T	6
STAT1	T	F	T	F	F	T	T	F	T	5
TRFRSF10C	T	T	F	T	F	F	F	T	T	5
PARP1	T	F	T	F	T	T	T	F	F	5
CXCL8	F	T	F	T	F	F	T	T	T	5
IL1A	F	T	F	T	F	T	F	T	T	5
CXCL1	F	T	F	T	F	T	F	T	T	5
ICAM1	F	T	T	T	F	F	F	T	T	5
CCL2	F	T	F	T	F	T	F	T	T	5
IGFBP2	F	T	F	T	T	F	F	T	T	5
AXL	F	T	F	T	F	T	F	T	T	5
WNT2	F	T	F	T	F	T	F	T	T	5

HMGB2	F	T	T	F	T	T	F	F	T	5
HMGB1	F	T	T	T	T	T	F	F	F	5
IGFBP5	F	F	T	T	F	T	T	F	T	5
GDF15	F	F	T	T	F	T	F	T	T	5
MDM2	F	F	T	F	T	T	T	F	T	5
CDKN2B	F	F	T	F	T	T	T	F	T	5
CCNA2	F	F	T	F	T	T	T	F	T	5
CDK1	F	F	T	F	T	T	T	F	T	5
HELLS	F	F	T	F	T	T	T	F	T	5
FOXM1	F	F	T	F	T	T	T	F	T	5
BUB1B	F	F	T	F	T	T	T	F	T	5
LMNB1	F	F	T	F	T	T	T	F	T	5
BRCA1	F	F	T	F	T	T	T	F	T	5
IGF1	F	F	F	T	T	F	T	T	T	5
JUN	F	F	F	T	T	T	T	T	F	5
MIF	F	F	F	T	T	T	T	T	F	5
TGFB1	F	F	F	T	T	F	T	T	T	5

T, true; F, false.

Table S6. Salivary Secretion-related Gene Sets.

Activity	Channel protein	Gene
Ca^{2+} channel	TRPC1	TRPC1
Ca^{2+} channel	TRPC3	TRPC3
Ca^{2+} channel	Orai1	ORAI1
Ca^{2+} channel	Orai2	ORAI2

Ca^{2+} channel	Orai3	ORAI3
K^+ channel	KCa1.1	KCNMA1
K^+ channel	KCa3.1	KCNN4
Cl^- channel	TMEM16A	ANO1
Na^+/H^+ exchanger	NHE1	SLC9A1
Cl^-/HCO^{3-} exchanger	AE2	SLC4A2
Na^+-K^+-$2Cl^-$ Co-transporter	NKCC1	SLC2A2
Na^+/K^+ ATPase	Alpha 1 Na^+/K^+ ATPase	ATP1A1
Water channel	Aquaporin 1	AQP1
Water channel	Aquaporin 3	AQP3
Water channel	Aquaporin 4	AQP4
Water channel	Aquaporin 5	AQP5
Water channel	Aquaporin 6	AQP6
Water channel	Aquaporin 7	AQP7
Water channel	Aquapori8	AQP8
Water channel	Aquaporin 11	AQP11

Table S7. Multivariable linear regression analyses of salivary gland focus score

Outcome	Variable	Standardized β	*P* value	VIF
Focus score	GZMK IHC score	**0.394**	**0.030**	1.905
	Age	−0.092	0.537	1.360
	Sex	−0.010	0.941	1.097
	Anti-SSA/SSB antibody	**0.483**	**0.003**	1.410
	Xerostomia duration	−0.192	0.182	1.254

$R^2 = 0.460$, adjusted $R^2 = 0.380$, $F_{5,34} = 5.787$, $P = 0.001$.
